# Detection and discovery of plant viruses in soybean by metagenomic sequencing

**DOI:** 10.1186/s12985-022-01872-5

**Published:** 2022-09-13

**Authors:** Manjula G. Elmore, Carol L. Groves, M. R. Hajimorad, Tracey P. Stewart, Mikaela A. Gaskill, Kiersten A. Wise, Edward Sikora, Nathan M. Kleczewski, Damon L. Smith, Daren S. Mueller, Steven A. Whitham

**Affiliations:** 1 Department of Plant Pathology, Entomology, and Microbiology, Iowa State University, 2213 Pammel Drive, Ames, IA 50011-1101 USA; 2 Department of Plant Pathology, University of Wisconsin-Madison, Madison, WI 53706 USA; 3 Department of Entomology and Plant Pathology, University of Tennessee, Knoxville, TN 37996 USA; 4 Roy J. Carver High Resolution Microscopy Facility, Iowa State University, Ames, IA 50011 USA; 5 Department of Plant Pathology, University of Kentucky, Princeton, KY 43445 USA; 6 Department of Entomology and Plant Pathology, Auburn University, Auburn, AL 36849 USA; 7 Department of Crop Sciences, University of Illinois, Urbana, IL 61801 USA

**Keywords:** Soybean, High-throughput sequencing, Virus identification, Clover yellow vein virus, *Nicotiana benthamiana*, Broad bean, Mixed infection, Ilarvirus

## Abstract

**Background:**

Viruses negatively impact soybean production by causing diseases that affect yield and seed quality. Newly emerging or re-emerging viruses can also threaten soybean production because current control measures may not be effective against them. Furthermore, detection and characterization of new plant viruses requires major efforts when no sequence or antibody-based resources are available.

**Methods:**

In this study, soybean fields were scouted for virus-like disease symptoms during the 2016–2019 growing seasons. Total RNA was extracted from symptomatic soybean parts, cDNA libraries were prepared, and RNA sequencing was performed using high-throughput sequencing (HTS). A custom bioinformatic workflow was used to identify and assemble known and unknown virus genomes.

**Results:**

Several viruses were identified in single or mixed infections. Full- or nearly full-length genomes were generated for tobacco streak virus (TSV), alfalfa mosaic virus (AMV), tobacco ringspot virus (TRSV), soybean dwarf virus (SbDV), bean pod mottle virus (BPMV), soybean vein necrosis virus (SVNV), clover yellow vein virus (ClYVV), and a novel virus named soybean ilarvirus 1 (SIlV1). Two distinct ClYVV isolates were recovered, and their biological properties were investigated in *Nicotiana benthamiana*, broad bean, and soybean. In addition to infections by individual viruses, we also found that mixed viral infections in various combinations were quite common.

**Conclusions:**

Taken together, the results of this study showed that HTS-based technology is a valuable diagnostic tool for the identification of several viruses in field-grown soybean and can provide rapid information about expected viruses as well as viruses that were previously not detected in soybean.

**Supplementary Information:**

The online version contains supplementary material available at 10.1186/s12985-022-01872-5.

## Background

Soybean (*Glycine max* L. Merr.) is valued worldwide for the high levels of protein and oil in its seeds, which have many uses in food, animal feed products, industrial feedstocks, and biodiesel production [1]. Soybean production is challenged by diseases caused by numerous microbial pathogens such as bacteria, fungi, oomycetes, and viruses that reduce yield and/or seed quality [2–8]. More than 100 viruses are known to infect soybean, and of these, at least 46 have been detected in naturally occurring infections in fields [6, 9]. Some of these viruses, such as soybean mosaic virus (SMV) are globally distributed and threaten soybean production in many countries [10–12]. In contrast, bean pod mottle virus (BPMV) is recognized as a major soybean pathogen mainly in the United States of America (USA) [13, 14]. These and other viruses including tobacco streak virus (TSV), alfalfa mosaic virus (AMV), soybean dwarf virus (SbDV), and tobacco ringspot virus (TRSV) have been known for several decades to cause disease problems [3, 4, 6].

In addition to well-established viruses, soybean production is also threatened by new and emerging viruses. Soybean vein necrosis virus (SVNV) was first identified from diseased fields in 2008. Since its discovery, SVNV has been reported on soybean in many states in the USA, Canada, and Egypt [15–19]. There are also reports of well-known viruses such as peanut mottle virus (PeMoV) [20], bean yellow mosaic virus (BYMV) [21], and bean common mosaic virus [22], associated with soybean diseases in the field. Of particular interest is clover yellow vein virus (ClYVV), a potyvirus that is primarily known to cause important diseases in forage legumes [23]. ClYVV was recently reported in field-grown soybeans in South Korea, and a partial genome was found in an RNA sequencing study performed with soybean samples collected in Ohio, USA [24, 25]. Although soybean is not normally considered as a host to ClYVV, a recent report showed wild soybeans (*Glycine soja*) were susceptible to ClYVV in contrast to cultivated soybeans (*Glycine max*) that were resistant to the ClYVV isolates tested [26].

Identification of viruses in plants predominantly relies on visual assessments coupled with microscopy and immuno- or PCR-based assays [27]. These tests require prior knowledge about the candidate virus causing the disease, and therefore have no or limited utility in identifying unknown viruses and unexpected viruses [28]. High-throughput sequencing (HTS) does not rely on prior knowledge of viruses infecting plant samples [27, 29–32]. The application of HTS has facilitated the identification and diagnosis of viruses, including unknown viruses in large-scale disease surveys [33–35]. In addition, HTS has been extensively used for plant virome studies [36–40]. Furthermore, HTS was successfully used to identify viruses and other pathogens in soybean vegetative parts [25, 41, 42], seeds [43], or in arthropod vectors that transmit them to soybean [44].

In this work, we scouted soybean fields for plants with virus-like or unusual symptoms, extracted total RNA, and performed RNA sequencing followed by bioinformatic analyses to determine if viruses were present. A total of 135 samples were collected and sequenced over the 2016 to 2019 growing seasons. From 78 virus-containing samples, complete or nearly complete viral genomes were assembled, including TSV, AMV, TRSV, BPMV, SbDV, and SVNV, which are well known viruses infecting soybean in the USA. Surprisingly, ClYVV was identified in samples collected from Iowa, USA in two different years, and the isolates were recovered and confirmed to be infectious in soybean, *Nicotiana benthamiana*, and *Vicia faba*. In addition, a new ilarvirus, provisionally named soybean ilarvirus 1 ( SIlV1), was identified and confirmed by RT-PCR and Sanger sequencing. Finally, our results indicate that mixed virus infections of various combinations are common occurrences.

## Methods

### Sample collection and RNA extraction

**Fig. 1:**
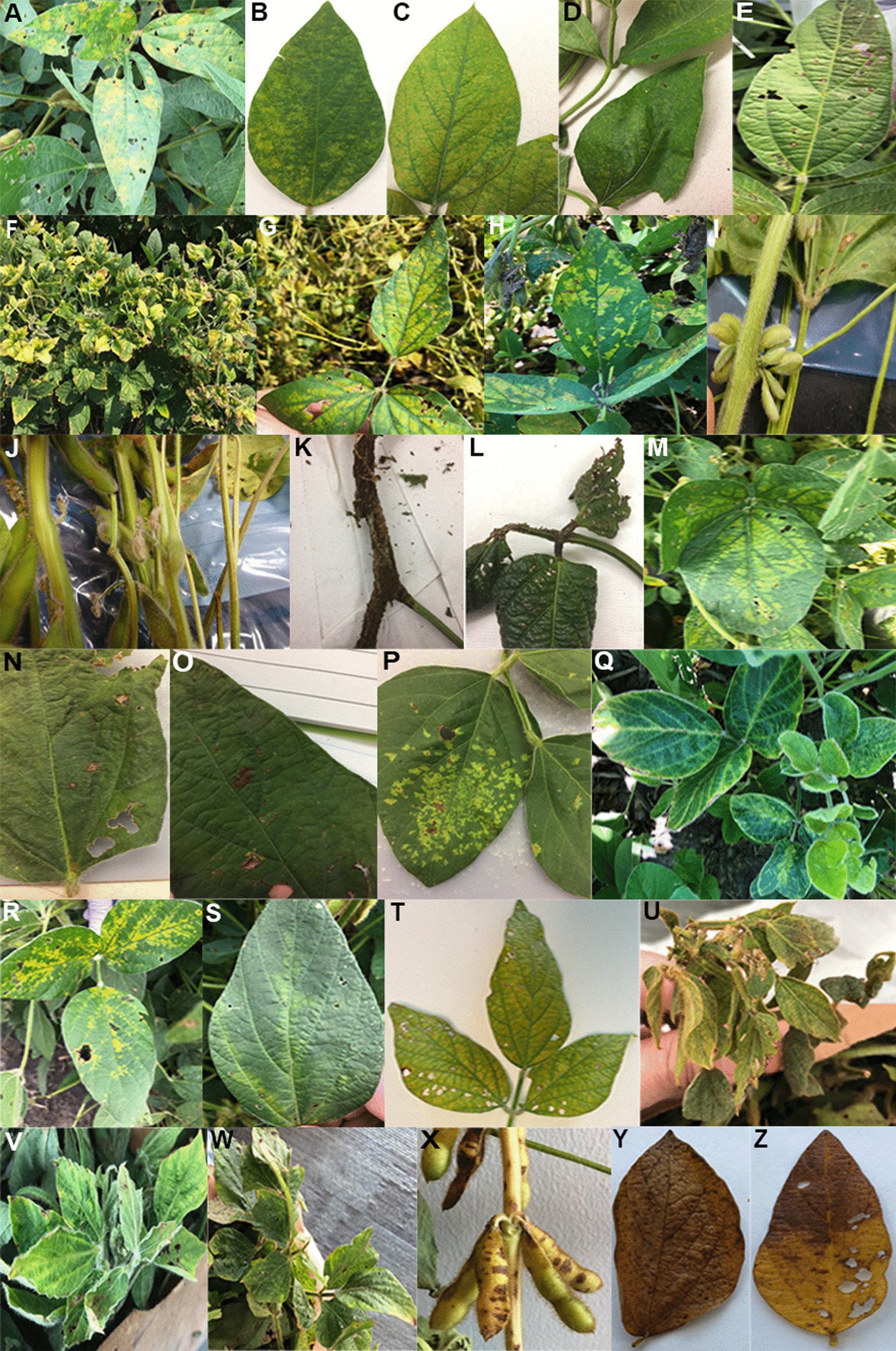
Representative images of symptomatic soybean tissues collected from the field for metagenomics analysis. **A**–**H** Leaves showing different patterns of chlorosis. A-H represent samples S1, S7, S12, S13, S18, S24, S25, and S26 respectively. **I**, **J** Examples of bud proliferation. I-J represent samples S4 and S14 respectively. **K** Stems thickened with brown corky necrosis. K represents sample S5. **L**, **M** Leaves exhibiting mottling. L-M represent samples S6 and S19 respectively. **N**, **O** Leaves with brown necrotic spots. N–O represent samples S39 and S40 respectively. **P** Leaves with angular yellow spots. P represents sample S41. **Q**–**S** Leaves displaying chlorosis of the veins or the tissue immediately surrounding the veins. Q-S represent samples S15, S16, and S17 respectively. **T**, **U** Leaves showing puckering and chlorosis. T-U represent samples S20 and S21 respectively. **V**, **W** Leaves exhibiting wilting and mosaic symptoms. V-W represent samples S22 and S23 respectively. **X** Pods with necrotic spots. X represent sample S24. **Y**, **Z** Leaves that rapidly became generally chlorotic and then necrotic. Y–Z represent samples S27, S28 respectively

A total of 135 soybean samples displaying virus-like or unusual disease symptoms were collected from eight different states in the USA during the 2016–2019 growing seasons and stored at −80 °C. Images of leaves from the 78 virus-containing samples that possessed one or more complete or nearly complete viral genome(s) are shown in Fig. 1 and Additional file 1: Fig. S1. A list of the 78 samples with their origin, description of symptoms, and associated complete or nearly complete viral genome(s) is provided in Table 1 and Additional files 11 and 12: Tables S1 and S2.

**Table 1:** Sample location, year collected, associated symptoms, and viruses identified with their assigned GenBank accession numbers in field-grown soybean across Iowa, USA

Sample ID	Iowa county	Year	Symptoms✝	Virus identified and GenBank accession number	Assigned name
S1	Story	2016	Chlorosis	Clover Yellow Vein Virus (ClYVV) [MK292120]	IA-2016
S2	Story	2016	Virus-like	Soybean dwarf virus (SbDV) [MT526793]	IA-2016
S3	Story	2016	Virus-like	Tobacco streak virus (TSV, RNA1) [MT596818] Tobacco streak virus (TSV, RNA2) [MT596819] Tobacco streak virus (TSV, RNA3) [MT596820]	IA-2016
S4	Marion	2017	Bud proliferation	Alfalfa mosaic virus (AMV, RNA1) [MT596806] Alfalfa mosaic virus (AMV, RNA2) [MT596807] Alfalfa mosaic virus (AMV, RNA3) [MT596808]	IA-2017
S5	No data	2017	Corky stem	Bean pod mottle virus (BPMV, RNA1) [MT521477] Bean pod mottle virus (BPMV, RNA2) [MT521478]	IA-2017
S6	No data	2017	Rugose leaves	Bean pod mottle virus (BPMV, RNA1) [MT669389] Bean pod mottle virus (BPMV, RNA2) [MT669390]	IA-2-2017
S7	Washington	2017	mosaic, vein clearing	Clover Yellow Vein Virus (ClYVV) [MK318185]	IA-2017
S8	No data	2017	Virus-like	Soybean dwarf virus (SbDV) [MT526794]	IA-2017
S9	No data	2017	Virus-like	Tobacco streak virus (TSV, RNA1) [MT602528] Tobacco streak virus (TSV, RNA2) [MT602529] Tobacco streak virus (TSV, RNA3) [MT602530]	IA-1-2017
S10	Story	2017	leaf puckering	Tobacco streak virus (TSV, RNA1) [MT602531] Tobacco streak virus (TSV, RNA2) [MT602532] Tobacco streak virus (TSV, RNA3) [MT602533]	IA-2-2017
S11	No data	2017	Virus-like	Tobacco streak virus (TSV, RNA1) [MT669383] Tobacco streak virus (TSV, RNA2) [MT669384] Tobacco streak virus (TSV, RNA3) [MT669385]	IA-3-2017
S12	No data	2017	Chlorosis, mottling	Tobacco ringspot virus (TRSV, RNA1) [MT563078] Tobacco ringspot virus (TRSV, RNA2) [MT563079]	IA-1-2017
S13	No data	2017	Mottling, stunted plant	Tobacco ringspot virus (TRSV, RNA1) [MT563080] Tobacco ringspot virus (TRSV, RNA2) [MT563081]	IA-2-2017
S14	Lee	2017	Bud proliferation	Tobacco ringspot virus (TRSV, RNA1) [MT563082] Tobacco ringspot virus (TRSV, RNA2) [MT563083]	IA-3-2017
S15	Story	2018	Brown vein and necrosis	Alfalfa mosaic virus (AMV, RNA1) [MT596809] Alfalfa mosaic virus (AMV, RNA2) [MT596810] Alfalfa mosaic virus (AMV, RNA3) [MT596811]	IA-1-2018
S16	Boone	2018	Chlorosis, especially near the veins	Alfalfa mosaic virus (AMV, RNA1) [MT596812] Alfalfa mosaic virus (AMV, RNA2) [MT596813] Alfalfa mosaic virus (AMV, RNA3) [MT596814]	IA-2-2018 implePara>
S17	Boone	2018	Chlorosis, especially near the veins	Alfalfa mosaic virus (AMV, RNA1) [MT596815] Alfalfa mosaic virus (AMV, RNA2) [MT596816] Alfalfa mosaic virus (AMV, RNA3) [MT596817]	IA-3-2018
S18	Boone	2018	Chlorosis, rugose	Alfalfa mosaic virus (AMV, RNA1) [MT669391] Alfalfa mosaic virus (AMV, RNA2) [MT669392] Alfalfa mosaic virus (AMV, RNA3) [MT669393]	IA-4-2018
S19	Boone	2018	Interveinal chlorosis	Bean pod mottle virus (BPMV, RNA1) [MT526791] Bean pod mottle virus (BPMV, RNA2) [MT526792]	IA-2018
S20	Story	2018	Interveinal chlorosis	Soybean dwarf virus (SbDV) [MT669394]	IA-1-2018
S21	Story	2018	Interveinal chlorosis	Soybean dwarf virus (SbDV) [MT669395]	IA-2-2018
S22	Story	2018	Chlorosis, puckering	Tobacco streak virus (TSV, RNA1) [MT602534] Tobacco streak virus (TSV, RNA2) [MT602535] Tobacco streak virus (TSV, RNA3) [MT602536]	IA-1-2018
S23	Story	2018	Chlorosis, puckering	Tobacco streak virus (TSV, RNA1) [MT602537] Tobacco streak virus (TSV, RNA2) [MT602538] Tobacco streak virus (TSV, RNA3) [MT602539]	IA-2-2018
S24*	Story	2018	Chlorosis, early senescence, necrosis on pods	Alfalfa mosaic virus (AMV) Tobacco streak virus (TSV)	–
S25*	Story	2018	Interveinal chlorosis	Alfalfa mosaic virus (AMV) Tobacco streak virus (TSV)	–
S26*	Hancock	2018	Chlorotic patches between veins	Alfalfa mosaic virus (AMV) Tobacco streak virus (TSV)	–
S27	Tama	2019	Yellowing and death of upper young leaves	Tobacco streak virus (TSV, RNA1) [MT602540] Tobacco streak virus (TSV, RNA2) [MT602541] Tobacco streak virus (TSV, RNA3) [MT602542]	IA-1-2019
S28	Story	2019	Yellowing and death of upper young leaves	Tobacco streak virus (TSV, RNA1) [MT602543] Tobacco streak virus (TSV, RNA2) [MT602544] Tobacco streak virus (TSV, RNA3) [MT602545]	IA-2-2019
S78	Story	2016	Virus-like	Soybean ilarvirus 1 (SIlV1, RNA1) [OL539723] Soybean ilarvirus 1 (SIlV1, RNA2) [OL539724] Soybean ilarvirus 1 (SIlV1, RNA3) [OL539725]	IA-2016

*Indicates co-infections

^†^Samples lacking detailed symptom description or images are denoted as ‘Virus-like’

Samples collected in 2016 were ground in liquid nitrogen and total RNA was extracted using the Pure Link™ RNA Mini-Kit (Invitrogen by Fisher Scientific, Carlsbad, CA). Approximately 100 mg of leaf tissue ground in liquid nitrogen was added to Trizol and mixed thoroughly before being processed according to the manufacturer’s directions. Total RNA was treated with DNase I (TURBO DNA-free™ Kit, ThermoFisher Scientific) to remove genomic DNA contamination. The 2017–2019 samples were ground in liquid nitrogen and total RNA was extracted using Direct-zol RNA Miniprep Plus (www.zymoresearch.com) that included an on-column DNAse treatment following the manufacturer’s instructions. Total RNA was quantified with a Qubit 4.0 fluorometer (Life Technologies, Carlsbad, CA, USA) and quality was assessed with a NanoDrop 2000 (ThermoFisher Scientific, CA, USA). Samples with high quality total RNA had A260/280 and A260/230 ratios between 1.8 and 2.1. Samples with low quality total RNA had A260/280 and A260/230 ratios between 1.0 and 1.8.

### cDNA library preparation and RNA sequencing

The DNA-free total RNA was ribosome depleted (Ribo-Zero rRNA Removal Kit (Plant Leaf), Illumina, San Diego, CA, USA; RiboMinus Plant kit, ThermoFisher Scientific, CA, USA). Strand-specific cDNA libraries were prepared from ribosome-depleted RNA using the NEBNext Ultra II RNA library prep kit (New England Biolabs, Ipswich, MA, USA) [45]. Paired-end Illumina sequencing was performed on the dual-indexed cDNA libraries (New England Biolabs, Ipswich, MA) using HiSeq3000 (150 bp from each end; 2 lanes per library; Illumina, San Diego, CA; Additional file 2: Fig. S2A).

### Bioinformatics workflow

FastQC v.0.11.2 was used to assess the quality of the raw sequence reads to determine the necessity of trimming low-quality reads [46]. All paired-end FASTQ files were processed with Trimmomatic v0.36 [47] using sliding window 4:15 and excluding reads below a minimal length of 36. The trimmed paired-end FASTQ sets were examined by FastQC again to confirm improvement of read quality. Trimmed paired-end RNA-Seq reads were mapped against the soybean genome v2.1 (https://plants.ensembl.org/Glycine_max) [48] using the HISAT2 alignment program [49] (–un-conc –al-conc). The reads with sequence similarities greater than 60% to the soybean genome sequences were removed. The remaining unmapped reads were mapped to plant virus reference genome sequences using Bowtie2 aligner [50] (–sensitive-local –un-conc –al-conc). The plant virus reference sequences were composed of 20,433 virus genome sequences obtained from the NCBI GenBank (NCBI: National Center for Biotechnology Information; https://www.ncbi.nlm.nih.gov/genome/viruses). The mapped viral reads and the unmapped non-soybean non-viral reads were de novo assembled separately using Trinity v2.6.6 [51] with a default k-mer of 25 to obtain potential viral contigs. All assembled contigs were queried against the all-organism NCBI nucleotide and protein database through BLASTN (Basic Local Alignment Search Tool) and BLASTX searches with default parameters using Blastplus v2.7.1 (Additional file 2: Fig. S2B) [52, 53]. A virus was determined as present in each sample if at least one contig had significant hits (E-value < 1e-10 for BLASTN, and E-value < 1e-5 for BLASTX as cutoff) to NCBI sequences with the virus description and with a query coverage greater than 80% and identity greater than 95%. Contigs matching bacteria, fungi or insect sequences with high query coverage and high sequence identity were removed. Contigs less than 1000 bp matching viruses with lower query coverage and sequence identity were not considered.

### Phylogenetic analysis

A phylogenetic tree was constructed from the alignment of two ClYVV IA isolates (IA-2016 and IA-2017), and other potyvirus species using PhyML 3.0 software [54]. In brief, the amino acid sequences of the polyprotein from the two identified ClYVV IA isolates, nine known ClYVV isolates from GenBank, and as an outgroup, two bean yellow mosaic virus (BYMV) isolates were aligned using MUSCLE alignment software [55]. Additionally, the phylogenetic analysis of SIlV1 was conducted using the amino acid sequences of movement protein (MP), coat protein (CP), RNA dependent RNA polymerase (RdRp) and replicase obtained in this study and other established ilarvirus species using PhyML 3.0 software. The full-length genome sequences of SIlV1 were submitted to ORF finder [56] to determine the predicted amino acid sequences. Alignments were performed using MUSCLE 3.8.31 alignment software [55]. To construct phylogenetic trees for both ClYVV and SIlV1 isolates, maximum likelihood analysis was performed using PhyML 3.0 phylogeny online server, with the bootstrap replication (1000 replicates) used to assess the statistical support of the groups on the tree. The best-fit substitution model used in the analysis was determined with the automatic model selection by Smart Model Selection (SMS [57],) on PhyML 3.0, using the Akaike Information Criterion (AIC, [58]) with the + G, + I, and + F parameters. FigTree v.1.4.4 (http://tree.bio.ed.ac.uk/software/figtree/) was used to visualize the phylogenetic tree.

### Virus preparation and plant inoculations

*N. benthamiana* were grown in a growth chamber set to 22/18 °C (day/night) with a 16/8 h light/dark photoperiod. Broad bean (*Vicia faba* cv. Broad Windsor and cv. Robin Hood; https://territorialseed.com) and soybean (Acre Edge 22R269, Williams 82, and 41 Nested Association Mapping (NAM) parents) seeds were germinated in growth chambers set to 28/24 °C (day/night) with a 16/8 h light/dark photoperiod. After ten days, seedlings were moved to a greenhouse kept at 28/24 °C (day/night) with a 16/8 h light/dark photoperiod. Three to four leaves of four-week-old *N. benthamiana*, or the primary leaves of ten-day old broad bean and soybean were lightly dusted with carborundum powder. Inoculum was prepared by grinding frozen tissue with a mortar and pestle in 10 mM phosphate buffer, pH 7.5. Leaves were rub-inoculated using a cotton swab. For mock-inoculated control plants, leaves were lightly dusted with carborundum and rub-inoculated with 10 mM phosphate buffer, pH 7.5. Three biological replicates were performed, and for ClYVV experiments, each replicate had seven plants (*N. benthamiana*), six plants (broad bean) or 20 plants (soybean). For TRSV experiments, each replicate included 20 plants. Symptomatic and mock-inoculated *N. benthamiana*, broad bean (cv. Broad Windsor and cv. Robin Hood), and soybean leaves were collected at 21 dpi and stored at −80 °C. Symptomatic soybean leaves from ClYVV IA-2016 plants and mock-inoculated leaves were collected at 35 dpi and stored at −80 °C.

### cDNA synthesis and sequence validation of annotated virus fragments

Reverse transcription-polymerase chain reaction (RT-PCR) was performed for the following samples: Field-grown soybean samples (S1-S78); greenhouse-grown *N. benthamiana* leaf tissues, broad bean leaf tissues, and soybean leaf tissues infected with ClYVV-IA-2016 or ClYVV-IA-2017; and greenhouse-grown *N. benthamiana* leaf tissues and soybean leaf tissues infected with TRSV. For greenhouse experiments, mock-inoculated *N. benthamiana* leaves, broad bean leaves, and soybean leaf tissues were used as negative controls. In brief, total RNA was extracted using Direct-zol RNA Miniprep Plus kit (www.zymoresearch.com) that included an on-column DNase treatment. Total RNA quality was assessed with a NanoDrop 2000 (ThermoFisher Scientific, CA, USA) and quantified with a Qubit 4.0 fluorometer (Life Technologies, Carlsbad, CA, USA). All RNA samples were reverse-transcribed using the RevertAid First Strand cDNA Synthesis Kit (ThermoFisher Scientific, CA, USA). The first-strand cDNA was quantified using Qubit 4.0 fluorometer. One µl of cDNA template was used in PCR carried out in 2X GoTaq Green Master Mix (Promega, USA), 10uM of forward and reverse primers in a total volume of 25ul. Amplifications were in a C1000 touch thermal cycler (Bio-Rad, Hercules, CA, USA) programmed as follows: 2 min at 95 °C with 32 cycles of 30 s at 94 °C, 30 s at 60 °C, and 45 s at 72 °C, followed by 10 min at 72 °C. The PCR products were visualized by electrophoresis on a 1% agarose gel containing SYBR Safe DNA gel stain (Life Technologies, Carlsbad, CA, USA). The PCR products were cleaned using ExoSAP-IT PCR product cleanup reagent (ThermoFisher Scientific, CA, USA) and sequenced by Sanger sequencing (DNA Facility, Iowa State University, Iowa, USA). For the new ilarvirus identified in sample (S78), PCR was performed with RNA1, RNA2, and RNA3 primer pairs under the following conditions: 2 min at 94 °C with 40 cycles of 1 min at 95 °C, 1 min at 54.5 °C, and 1 min at 72 °C, followed by 10 min at 72 °C. The PCR products were visualized by electrophoresis on a 1% agarose gel and gel purified using Wizard SV gel and PCR clean-up system (Promega, USA). The purified PCR products were sequenced by Sanger sequencing (University of Wisconsin Biotechnology Center, Madison, USA). Primer sequences were designed using Primer3 plus and PrimerQuest™ (www.idtdna.com) from assembled contigs, and the primers [43, 59–62] used in this study are listed in the Additional file 13: Table S3.

### Serological testing

Leaf pieces (~ 150 mg/sample) were macerated within a sterile pouch with buffer and then virus was detected using the ImmunoStrip for Potyvirus Group (Poty) (Agdia, Elkhart, IN). A positive or negative result was recorded within 20 min, and all individual strips were preserved. This ImmunoStrip was used to confirm the presence of potyvirus (ClYVV IA-2016 and ClYVV IA-2017) in each batch of *N. benthamiana* inoculum prior to inoculation on broad bean or soybean leaves. The ImmunoStrip was also used to confirm the presence or absence of potyviruses in soybean parents infected with ClYVV and corresponding progeny seedlings.

### ClYVV seed transmission tests

Seed transmissibility was examined using seeds collected from five greenhouse-grown ClYVV IA-2017-infected soybean plants (cv. Acre Edge 22R269) exhibiting disease symptoms. For controls, seeds were also collected from two mock-inoculated soybean plants (cv. Acre Edge 22R269). For seed transmission testing, 100 seeds from each ClYVV infected parent and mock-inoculated control parent plant were sown in growth chambers set to 26/24 °C (day/night) with a 16/8 h light/dark photoperiod. After fourteen days, two young leaflets from each soybean seedling were freshly harvested. A pool of leaflets from 10 soybean seedlings was harvested as an individual group. The parents prior to seed collection and the seedlings in groups of 10 for a total of 10 groups from each ClYVV-infected parent were serologically tested for presence of a potyvirus using the ImmunoStrip for Potyvirus Group as described above.

### Transmission electron microscopy (TEM)

*N. benthamiana* leaf tissues infected with ClYVV IA-2017 and corresponding leaf tissues of mock-inoculated plants were collected at 10 dpi and examined using TEM. Infected leaves were dissected and 2 mm portions were placed into 1% paraformaldehyde, 3% glutaraldehyde in 0.1 M sodium cacodylate buffer, pH 7.2 and fixed for 48 h at 4 °C. Samples were washed in 0.1 M cacodylate buffer three times/10 min each, and post fixed with 1% osmium tetroxide in 0.1 M sodium cacodylate buffer for 1 h at room temperature. Samples were washed with deionized water three times/15 min each, and *en bloc* stained using 2% uranyl acetate in distilled water for 1 h. Samples were washed in distilled water for 10 min and dehydrated through a graded ethanol series (25, 50, 70, 85, 95, and 100%) for 1 h each step. Samples were further dehydrated with three changes of pure acetone, 15 min each, and infiltrated with Spurr's formula (hard) epoxy resin (Electron Microscopy Sciences, Hatfield PA) with graded ratios of resin to acetone until fully infiltrated with pure epoxy resin (3:1, 1:1, 1:3, pure) for 6–12 h per step. Tissues were placed into BEEM capsules and were polymerized at 70 °C for 48 h. Thin sections were made using a Leica UC6 ultramicrotome (Leica Microsystems, Buffalo Grove, IL) at 50 nm and collected onto single slot carbon film grids. TEM images were collected using a 200 kV JEOL JSM 2100 scanning transmission electron microscope (Japan Electron Optics Laboratories, USA, Peabody, MA) with a GATAN One View 4 K camera (Gatan Incorporated, Pleasanton, CA).

## Results

### Collection and processing of field-grown soybean samples for HTS

Soybean samples with virus-like symptoms or disease symptoms of unknown etiology were collected for RNA sequencing during four growing seasons (2016–2019) as shown in Fig. 1, Table 1, Additional file 1: Fig. S1 and Additional files 11 and 12: Tables S1 and S2. The cDNA libraries generated from each sample were sequenced on an Illumina HiSeq 3000 platform (Additional file 2: Fig. S2A). The number of paired-end reads per sample ranged from 19,351,404 in S1 to 18,867,866 in S78, and after quality filtering the number of high quality trimmed raw reads ranged from 18,581,716 in S1 to 18,216,248 in S78 as shown in Additional file 14: Table S4.

### Mapping, de novo genome assembly, and identification of viruses

The filtered reads were mapped against the soybean genome v2.1 to remove soybean reads (Additional file 2: Fig. S2B) from the analyses as shown in Additional file 14: Table S4. The non-soybean reads were mapped to plant virus reference genome sequences (20,433 genome sequences; Additional file 2: Fig. S2B) obtained from NCBI GenBank as shown in Additional file 14: Table S4. The mapped viral reads were de novo assembled with a default k-mer of 25 using the Trinity assembler (Additional file 2: Fig. S2B). The unmapped non-soybean non-viral reads were also de novo assembled with a default k-mer of 25 using the Trinity assembler. To identify virus-associated sequences, the de novo assembled contigs were annotated against the all-organism NCBI nucleotide and protein database through BLASTN and BLASTX searches. Of the 135 samples, 78 of them (S1–S78) contained contigs corresponding to full-length or nearly full-length viral genomes and proteins as shown in Additional file 15: Table S5. Of the remaining 57 samples, 27 samples had de novo contig length less than 1000 bp with few hits to known viruses and other pathogens such as bacteria, oomycetes, and fungi. None of these samples had partial viral genomes. Additionally, 23 samples had no hits to viral genome sequence but uncovered pathogens such as bacteria, oomycetes, and fungi while the remaining 7 samples had no pathogens detected. Due to potentially misleading interpretations of partial sequences for virus identity [63], a conservative approach of only contigs corresponding to complete or nearly complete genome sequences were used in subsequent analyses. The top GenBank accessions that had the highest query coverage (> 80%) and highest sequence identity (> 95%) to each contig is listed in Additional file 15: Table S5. These contigs share similarity to SbDV, AMV, TSV, TRSV, BPMV, SVNV, and ClYVV viruses in the following families: *Luteoviridae*, *Bromoviridae*, *Secoviridae*, *Bunyaviridae*, and *Potyviridae*.

### Clover yellow vein virus (ClYVV): Identification and phylogenetics

A most surprising outcome from the sequence analyses was the detection of ClYVV in samples from Iowa, USA collected in 2016 (Sample S1) and 2017 (Sample S7; Table 1). Single contigs covering nearly the entire ClYVV reference genome (Gm isolate; GenBank accession number: KF975894.1) were obtained from each sample as shown in Additional file 15: Table S5. The sequences of the 2016 ClYVV isolate (IA-2016; GenBank accession number MK292120.1) and the 2017 ClYVV isolate (IA-2017; GenBank accession number MK318185.1) were 9610 nucleotides (nt) and 9627 nt long, respectively (Table 1; Additional file 15: Table S5), and they share 96.5% nt identity with one another (Additional file 3: Fig. S3). The ClYVV IA-2016 and -IA-2017 isolates shared the greatest nt sequence identity to the ClYVV Gm isolate, 96.39% and 96.32%, respectively, from South Korea (Additional file 15: Table S5). The ClYVV-IA-2016 and -IA-2017 genomes encode predicted polyproteins of 3,072 amino acids (aa) that were > 98% identical to the ClYVV I89-1 reference sequence (GenBank accession number: BAT50981.1) (Additional file 15: Table S5). The presence of ClYVV in S1 and S7 field samples was verified by PCR amplification using detection primers designed from the assembled contigs as listed in Additional file 13: Table S3, followed by Sanger sequencing as shown in Additional file 4: Fig. S4.

**Fig. 2:**
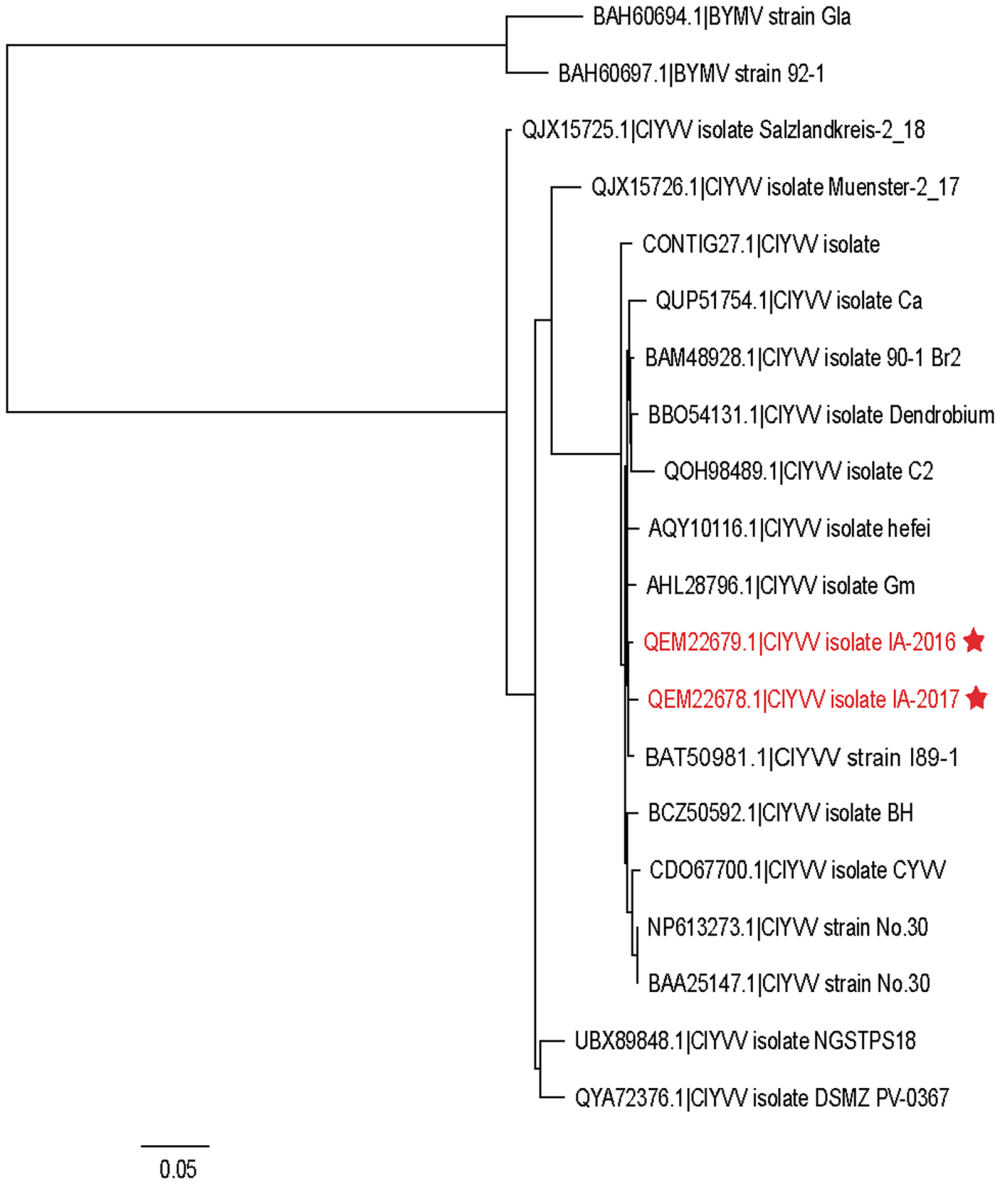
Maximum likelihood phylogenetic tree showing the relationship of the ClYVV-IA isolates to other ClYVV isolates and BYMV. The phylogenetic tree was based on the amino acid sequences of ClYVV and Bean yellow mosaic virus (BYMV). BYMV is included as an outgroup. This analysis used the fully sequenced genomes available in GenBank, except for ClYVV isolate contig27.1 Ohio USA, which was a partial sequence. Sequences were aligned using MUSCLE, and the tree constructed from the alignment using PhyML. The tree branches were bootstrapped with 1000 replications. The de novo assembled ClYVV-IA genomes identified in this study, along with their respective accession number are denoted by the red text and (*) star

To determine the relationships between the IA-2016 and IA-2017 isolates with the nine full-length ClYVV isolates in GenBank, the amino acid sequences were aligned, and a phylogenetic tree was constructed using the Maximum Likelihood algorithm. Sequences of two BYMV isolates from Japan were included as an outgroup, which only had 59.4–60.3% nt identity to ClYVV IA-2016 and ClYVV IA-2017. The ClYVV IA isolates formed a single cluster separate from China (Hefei isolate), South Korea (Dendrobium isolate), Ohio (contig 27.1), Japan (90-1 Br2 and No. 30 strains), and Australia ClYVV sequences (CYVV isolate; Fig. 2). ClYVV IA-2016 (MK292120.1) and ClYVV IA-2017 (MK318185.1) were most closely related to ClYVV I89-1 strain (BAT50981.1) and Gm isolate (AHL28796.1; Fig. 2), which is consistent with the nt sequence identity values shown in Additional file 15: Table S5. These results showed that ClYVV IA-2016 and ClYVV IA-2017 were distinct from one another, and they may have a common origin with isolates previously identified in Japan and South Korea.

### ClYVV infection and detection in *N. benthamiana*

**Fig. 3:**
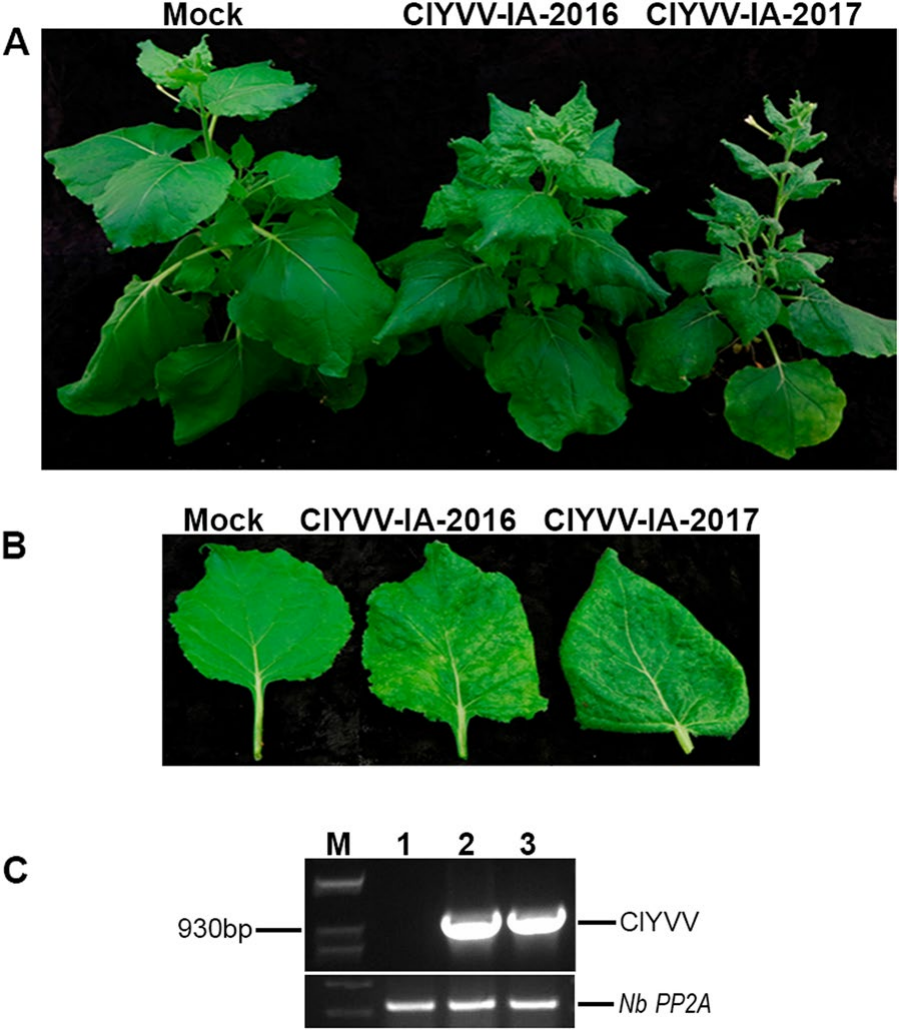
Symptoms of ClYVV-IA-2016 and ClYVV-IA-2017 in systemically infected leaves of *N. benthamiana*. Healthy mock-inoculated controls are indicated. Soybean field samples infected with ClYVV-IA-2016 or ClYVV-IA-2017 was used as an inoculum to infect *N. benthamiana* plants by mechanical inoculation. **A** Plants infected with ClYVV IA-2016 and ClYVV IA-2017 at 21 days post inoculation (dpi). **B** Systemic leaf with mosaic chlorosis and epinasty symptoms at 21 dpi. **C** RT-PCR detection of ClYVV isolates in *N. benthamiana* infected plants. Agarose gel electrophoresis of PCR confirming the presence of ClYVV-IA-2016 and ClYVV-IA-2017 identified by RNA sequencing. Lane 1: Mock- inoculated control; 2: Detection of ClYVV-IA-2016; 3: Detection of ClYVV-IA-2017; M: 1 Kb plus molecular weight maker. Sanger sequencing confirmed amplicon identities. *N. benthamiana* Protein phosphatase 2A (PP2A) reference gene was used as internal controls

To recover the ClYVV IA isolates, sap from the frozen soybean samples (S1 and S7) was used to mechanically inoculate *N. benthamiana* plants. At 10 dpi, systemic leaves of plants inoculated with either ClYVV IA-2016 or ClYVV IA-2017 exhibited mosaic chlorosis and epinasty (Additional file 5: Fig. S5). At 21 dpi, ClYVV IA-2017 plants were stunted, and the systemically-infected leaves showed pronounced curling and mosaic chlorosis (Fig. 3A and B). The ClYVV IA-2016 plants continued to exhibit milder symptoms (Fig. 3A and B). ClYVV IA-2016 and ClYVV IA-2017 in *N. benthamiana* plants was verified by PCR amplification (Fig. 3C) using detection primers designed from the assembled contigs (Additional file 13: Table S3) followed by Sanger sequencing (Additional file 6: Fig. S6).

### ClYVV isolates produce distinct symptoms in legumes

**Fig. 4:**
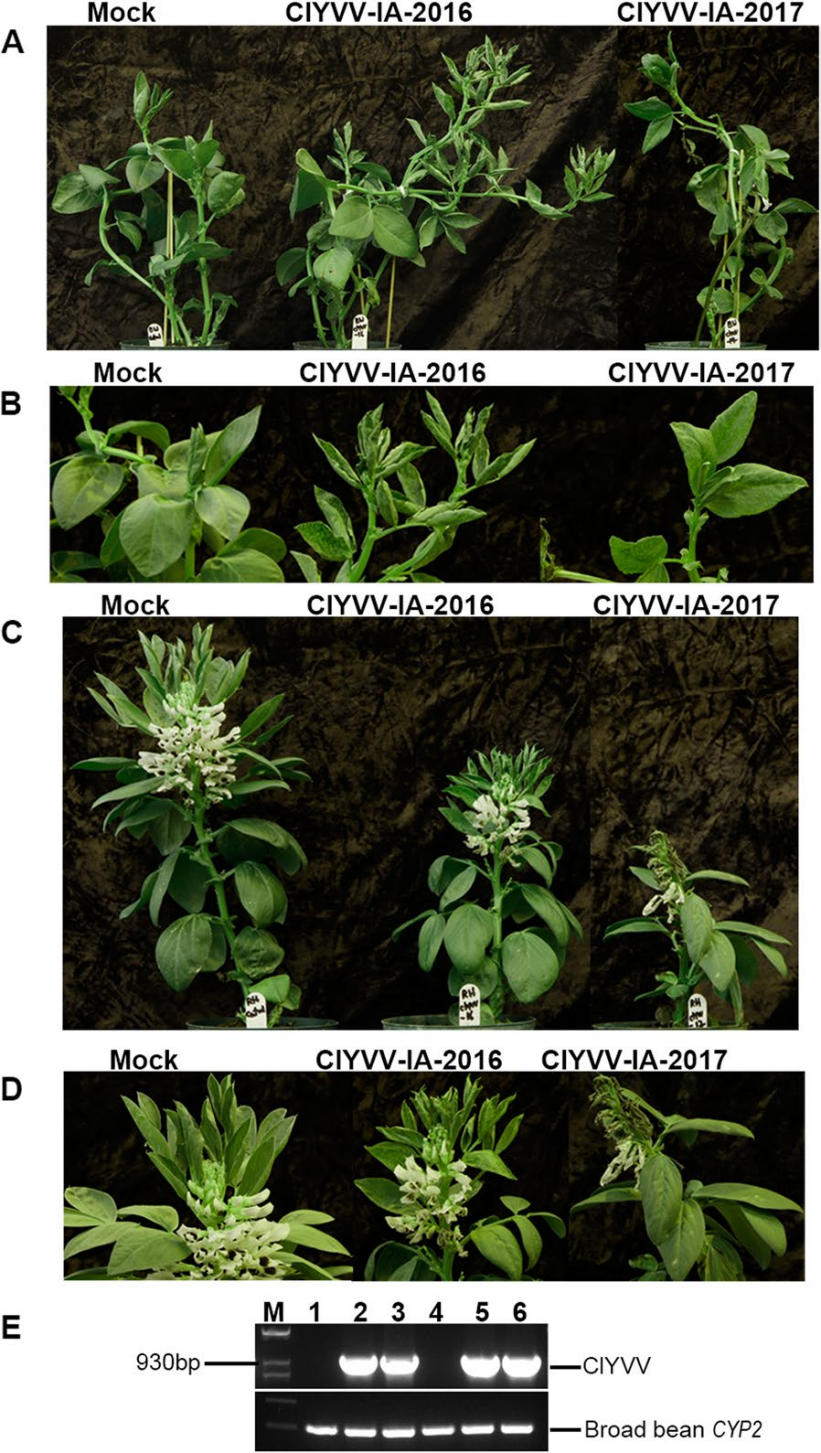
Symptoms caused by ClYVV-IA-2016 and ClYVV-IA-2017 in broad bean. Healthy mock-inoculated controls are indicated. *N. benthamiana* infected with ClYVV-IA-2016 or ClYVV-IA-2017 was used as an inoculum to infect broad bean plants by mechanical inoculation. **A** Whole plants (cv. Broad Windsor) infected with ClYVV IA-2016 and ClYVV IA-2017 at 21 days post inoculation (dpi). **B** Systemic leaves (cv. Broad Windsor) showing symptoms at 21 dpi. **C** Whole plants (cv. Robin Hood) infected with ClYVV IA-2016 and ClYVV IA-2017 at 21 dpi. **D** Systemic leaves (cv. Robin Hood) showing symptoms at 21 dpi. (**E**) RT-PCR detection of ClYVV isolates in broad bean infected plants. Agarose gel electrophoresis of PCR confirming the presence of ClYVV-IA-2016 and ClYVV-IA-2017 identified by RNA sequencing. Lanes 1 and 4: Mock- inoculated controls of Broad Windsor and Robin Hood respectively; 2 and 5: Detection of ClYVV-IA-2016 in Broad Windsor and Robin Hood respectively; 3 and 6: Detection of ClYVV-IA-2017 in Broad Windsor and Robin Hood respectively; M: 1 Kb plus molecular weight maker. Sanger sequencing confirmed amplicon identities. Broad bean cyclophilin (CYP2) reference gene was used as internal controls

To determine if the two ClYVV isolates systemically infect legumes, mechanical transmission was performed on broad bean and soybean plants. At 10 dpi, symptoms of mottling and hyponasty were observed on the systemic leaves of broad bean cultivars Broad Windsor and Robin Hood that were inoculated with either isolate. At 21 dpi, Broad Windsor plants infected with ClYVV IA-2016 developed more severe symptoms with pronounced mottling and leaf curling (Fig. 4A and B). In contrast, mild mottling with subtle leaf curling was observed in ClYVV IA-2017-infected systemic leaves (Fig. 4A and B). Robin Hood plants infected with either ClYVV IA-2016 or ClYVV IA-2017 were severely stunted with mild mottling patterns on systemic leaves (Fig. 4C and D). Systemic leaves infected with ClYVV IA-2016 exhibited mild hyponasty, and while ClYVV IA-2017 did not cause leaf curling, systemic leaves and stems developed necrosis and eventually wilted (Fig. 4C and D). ClYVV IA-2016 and ClYVV IA-2017 in Broad Windsor and Robin Hood infected plants was verified by PCR amplification (Fig. 4E) using detection primers designed from the assembled contigs (Additional file 13: Table S3) followed by Sanger sequencing (Additional file 6: Fig. S6).

**Fig. 5:**
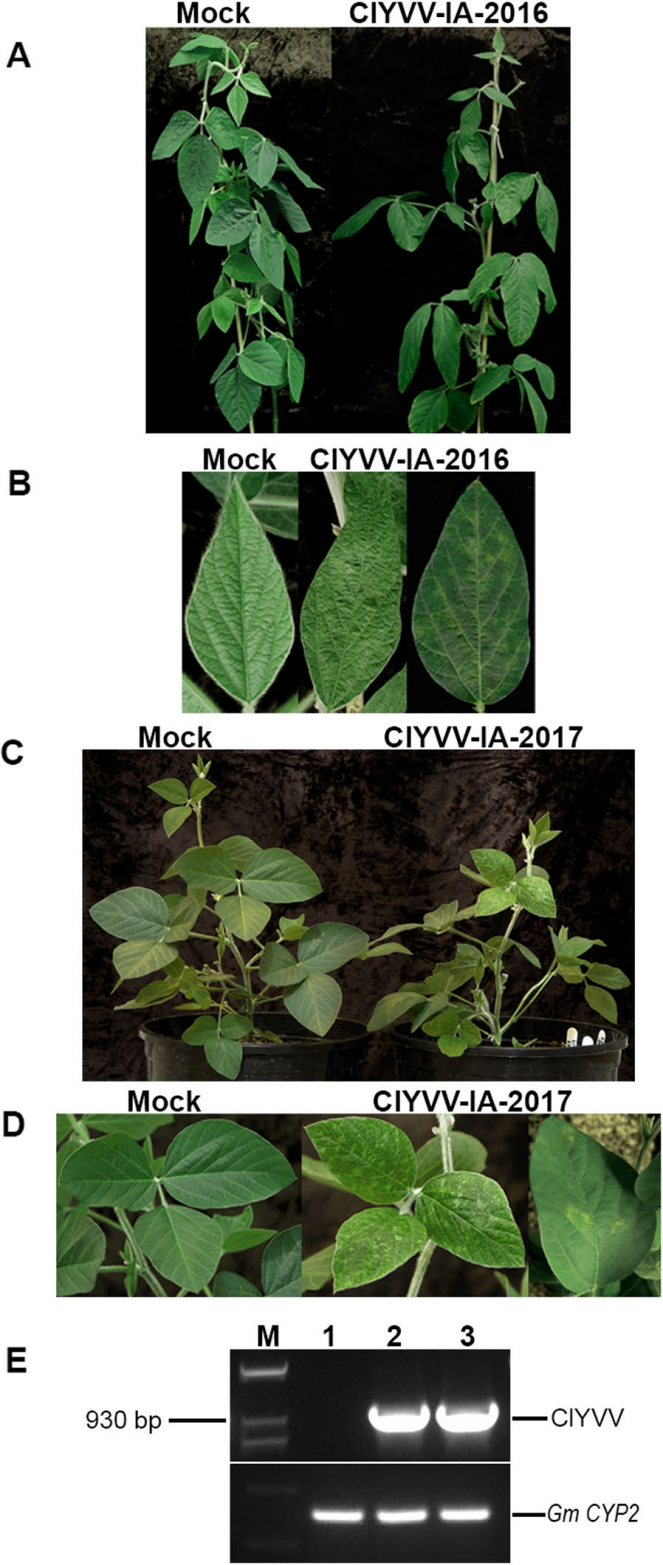
Symptoms caused by ClYVV-IA-2016 and ClYVV-IA-2017 in soybean. Healthy mock-inoculated controls are indicated. *N. benthamiana* infected with ClYVV-IA-2016 or ClYVV-IA-2017 was used as an inoculum to infect soybean plants by mechanical inoculation. **A** Whole plants infected with ClYVV IA-2016 at 35 days post inoculation (dpi). **B** Systemic leaf symptoms at 35 dpi. A representative young leaflet is shown in the middle of the panel while mature older leaflet is shown on the right. **C** Whole plants infected ClYVV IA-2017 at 21 dpi. **D** Systemic leaf symptoms at 21 dpi. A representative young trifoliolate leaf is shown in the middle of the panel while a mature older leaflet is shown on the right. **E** RT-PCR detection of ClYVV isolates in soybean infected plants. Agarose gel electrophoresis of PCR confirming the presence of ClYVV-IA-2016 and ClYVV-IA-2017 identified by RNA sequencing. Lane 1: Mock- inoculated control; 2: Detection of ClYVV-IA-2016; 3: Detection of ClYVV-IA-2017; M: 1 Kb plus molecular weight maker. Sanger sequencing confirmed amplicon identities. Soybean cyclophilin (CYP2) reference gene was used as internal controls

To determine infectivity in soybean, the cultivars Acre Edge 22R269 and Williams 82, and the 41 genetically diverse soybean lines contributing to the nested association mapping (NAM) panel were mechanically inoculated with ClYVV IA-2016 or ClYVV IA-2017. Acre Edge 22R269 was used in these experiments because it was the variety in which ClYVV IA-2016 was originally found. Chlorotic spots were observed at 30 dpi on older leaves of Acre Edge 22R269 plants infected with ClYVV IA-2016, which was followed by yellowing and vein clearing in older and younger systemic leaves by 35 dpi (Fig. 5A and B). Chlorotic spots were observed at 19 dpi on older leaves of Acre Edge 22R269 plants infected with ClYVV IA-2017, which was followed by yellowing and vein clearing in the older and younger systemic leaves by 21 dpi (Fig. 5C and D). ClYVV IA-2017-infected plants were stunted in growth, whereas ClYVV IA-2016-infected plants were not stunted in comparison to the mock-inoculated control plants (Fig. 5 A and C). ClYVV IA-2016 and ClYVV IA-2017 infection was verified in Acre Edge 22R269 plants by RT-PCR (Fig. 5E) using detection primers designed from the assembled contigs (Additional file 13: Table S3) followed by Sanger sequencing (Additional file 6: Fig. S6). Neither ClYVV isolate was able to infect Williams 82 or the 41 NAM parents. Additionally, Acre Edge 22R269 infected plants were used as a source of inoculum to infect Broad Windsor and Robin Hood plants. At 21 dpi, Robin Hood plants infected with either ClYVV IA-2016 or ClYVV IA-2017 were severely stunted with mottling patterns on systemic leaves (Additional file 7: Fig. S7A). Broad Windsor plants infected with ClYVV IA-2016 or ClYVV IA-2017 developed symptoms with mottling and leaf curling (Additional file 7: Fig. S7B). ClYVV IA-2016 and ClYVV IA-2017 in both broad bean cultivars was verified by RT-PCR amplification (Additional file 7: Fig. S7C) using detection primers designed from the assembled contigs (Additional file 13: Table S3) followed by Sanger sequencing (Additional file 6: Fig. S6).

### Electron microscopy of ClYVV-infected tissues

**Fig. 6:**
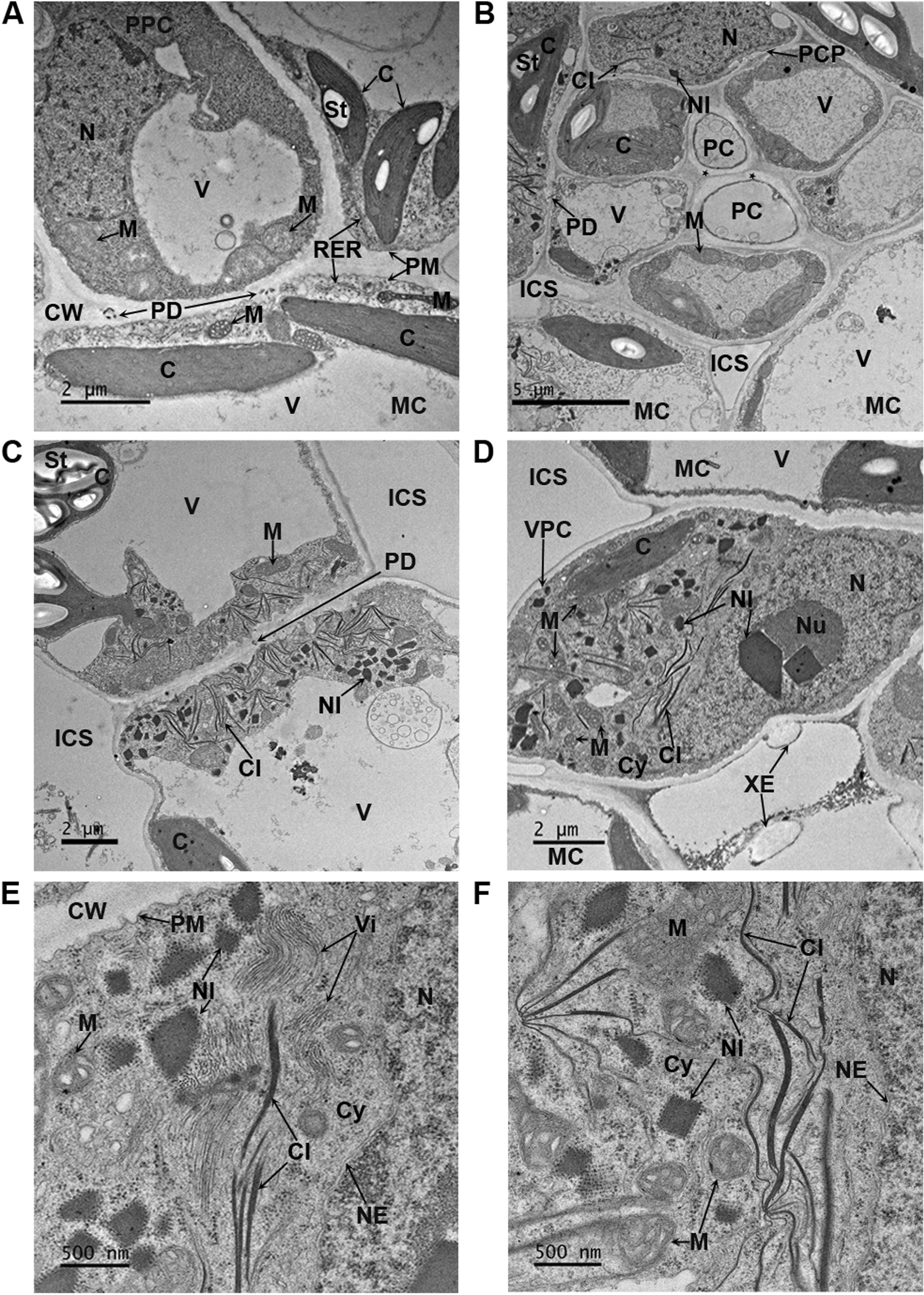
Transmission electron micrographs showing the ultrastructure of *N. benthamiana* mock-inoculated and ClYVV (IA-2017 isolate) infected leaf tissues **A** Mock inoculated leaf tissue at 10 dpi. Uninfected phloem parenchymal cell (PPC) surrounded by mesophyll cells (MC). Bar = 2 μm. (**B-F**) ClYVV infected leaf tissue at 10 dpi. **B** Infected vascular bundle surrounded by mesophyll cells (MC). Crystalline nuclear inclusions (NI) and cylindrical cytoplasmic inclusions (CI) distribution in the phloem cell parenchyma (PCP). Bar = 5 μm. **C** Infected mesophyll cells (MC). Crystalline nuclear inclusions (NI) and cylindrical cytoplasmic inclusions (CI) distribution in adjacent mesophyll cells. Bar = 2 μm. **D** Infected vascular parenchymal cell (VPC) above a xylem element (XE) and mesophyll cell (MC). Crystalline nuclear inclusions (NI) distribution in the nucleolus (Nu) and cytoplasm (Cy). Cylindrical cytoplasmic inclusions (CI) distribution in the cytoplasm. Bar = 2 μm. **E** Magnified virus particles from section of panel D. Virions (Vi), crystalline nuclear inclusions (NI) and cylindrical cytoplasmic inclusions (CI) distribution in the cytoplasm (Cy). Bar = 500 nm. **F** Magnified virus particles from section of panel D. Crystalline nuclear inclusions (NI) and cylindrical cytoplasmic inclusions (CI) distribution in the cytoplasm (Cy). Bar = 500 nm. C, chloroplast; St, starch; M, mitochondria; RER, rough endoplasmic reticulum; N, nucleus; NE, nuclear envelope; V, vacuole; CW, cell wall; PM, plasma membrane, PD, plasmodesmata; ICS, intracellular space; PC, phloem cell; Asterisk (*), phloem secondary cell wall thickening

Transmission electron microscopy (TEM) was performed to visualize ClYVV IA-2017-infected *N. benthamiana* cells at 10 dpi. Infected *N. benthamiana* tissues contained flexuous filamentous virus particles of 760–780 nm in length, and cytoplasmic and nuclear inclusions typical of those caused by ClYVV and other potyviruses [64]. ClYVV was observed in the phloem parenchyma and mesophyll cells of infected tissues. The cylindrical cytoplasmic inclusions comprising laminated aggregates induced by ClYVV were observed in the phloem parenchyma cells of the vascular bundle and surrounding mesophyll cells (Fig. 6B) but were absent in corresponding cells of mock-inoculated plants (Fig. 6A). Numerous cylindrical cytoplasmic inclusions with laminated aggregates were found in the cytoplasm of two adjacent mesophyll cells (Fig. 6C) and scattered throughout the cytoplasm of the vascular parenchymal cell located above a xylem element and mesophyll cell (Fig. 6D–F). The crystalline nuclear inclusions in the form of cuboidal or rhomboidal crystals induced by ClYVV was strongly associated within the nucleolus of the vascular parenchymal cell located above a xylem element and mesophyll cell (Fig. 6D). Additionally, several cuboid or rhomboid crystalline inclusions were observed in the cytoplasm of two adjacent mesophyll cells (Fig. 6C), phloem parenchyma cell 0 (Fig. 6B), and the vascular parenchymal cell located above a xylem element and mesophyll cell (Fig. 6D–F). Large areas containing virions aligned in parallel were observed in the cytoplasm of an infected vascular parenchymal cell (Fig. 6E). These results confirmed the presence of viable ClYVV identified in field-grown soybean.

### No evidence supporting ClYVV IA-2017 seed transmission

The potential for seed transmissibility of ClYVV IA-2017 was tested in Acre Edge 22R269. Seeds were collected from five ClYVV IA-2017-infected plants that were symptomatic and positive in the ImmunoStrip for Potyvirus Group assay (Additional file 8: Fig. S8A). Seeds were also collected from two mock-inoculated control plants. The 500 progeny seedlings tested from the five ClYVV-infected parents (P1 to P5) did not exhibit disease symptoms and appeared identical to the progeny seedlings from the mock-inoculated parents. For each parent, leaf samples from ten pools of ten seedlings were collected at 14 days after germination and assayed using the ImmunoStrip for Potyvirus Group (Additional file 8: Fig. S8B). Consistent with the lack of symptoms, all 50 pools tested negative for potyvirus, indicating that ClYVV-IA-2017 is either not seed transmissible, or seed transmission occurs at a frequency of less than 0.2% in Acre Edge 22R269.

### Specificity of ClYVV infection in soybean

Of the diverse soybean lines that were tested, Acre Edge 22R269 was unique in its ability to support systemic infection by ClYVV-IA-2016 and ClYVV-IA-2017. This led us to investigate whether Acre Edge 22R269 is generally susceptible to ClYVV. Acre Edge 22R269 seedlings were inoculated with ClYVV-No. 30 [65], soybean mosaic virus strain N (SMV-N) as a positive control, and two chimeric viruses ClYVV-No. 30 containing HC-Pro from SMV-N and SMV-N containing HC-Pro from ClYVV-No.30 [66]. The seedlings were susceptible to SMV-N and SMV-N containing the HC-Pro from ClYVV-No.30, but not to ClYVV-No. 30 or the chimeric ClYVV-No. 30 carrying the SMV-N HC-Pro (Additional file 16: Table S6). These results indicate that Acre Edge 22R269 is not generally susceptible to ClYVV, and further suggest that the host range determinant is not the HC-Pro protein [66].

### Novel ilarvirus identified in soybean

**Fig. 7:**
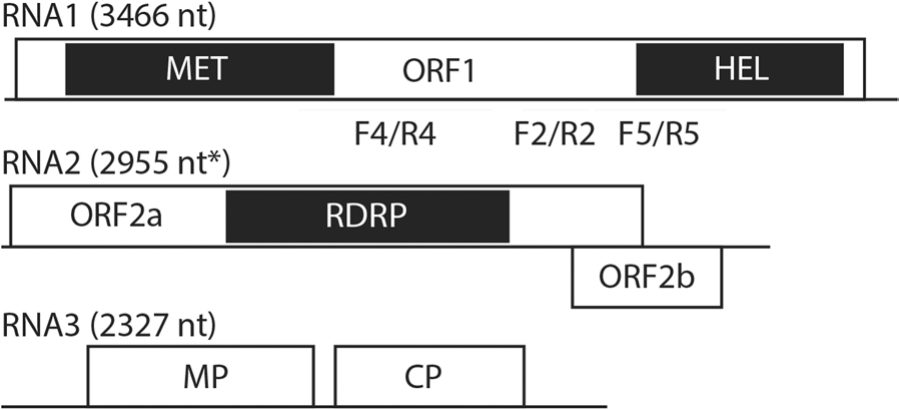
Genome organization of soybean ilarvirus 1 (SIlV1). The line segments indicate the genome sizes, and the boxes indicate each open reading frame (ORF). The RNA methyltransferase (MET), RNA helicase (HEL), and viral RNA dependent RNA polymerase (RDRP) domains were identified using CD-search [67] and are indicated by black shading. The asterisk indicates that RNA2 is likely lacking a few bases at the 5’ end and is not full-length. Although the termini were not confirmed by rapid amplification of cDNA ends, the termini of RNA1 and RNA3 obtained from our sequence assembly are consistent with the full-length sequences of related viruses

Three contigs (RNA1, RNA2 and RNA3 segments; Fig. 7) identified from sample S78 collected in Iowa, USA in 2016 had high sequence identity to various ilarviruses in BLASTN searches against the GenBank nr database. The RNA1 segment was approximately 75% identical to parietaria mottle virus (PMoV) (FJ858202.1, MZ405646.1, KT005243.1, AY496068.1), tomato necrotic spot virus (MH780154.1), and bacopa chlorosis virus (FJ607140.1). The RNA2 segment had the highest sequence coverage and 76% sequence identity to PMoV strain CR8 (FJ858203.1), and the RNA3 segment had the highest query coverage and 72% sequence identity to bacopa chlorosis virus (JQ015298.1). The tripartite genome organization is consistent with other ilarviruses (Fig. 7). RNA1 encodes a 1,092 aa protein containing viral RNA methyltransferase and RNA helicase domains (ORF1a), RNA2 encodes the RNA dependent RNA polymerase (RdRp; ORF2a, 812 aa) and the overlapping ORF2b (201 aa), and RNA3 encodes the movement protein (MP; 293 aa) and coat protein (CP; 246 aa). Because the highest nucleotide identity for any RNA segment was only 76%, we suggest that this is a novel *Ilarvirus*, for which we propose the name, soybean ilarvirus 1(SIlV1) IA-2016 isolate. PCR primer sets (Additional file 13: Table S3) were designed to different regions of RNA1, RNA2, and RNA3 to confirm the presence of this virus in S78, and the PCR amplicons were sequenced to confirm their identity (Additional file 4: Fig. S4).

**Fig. 8:**
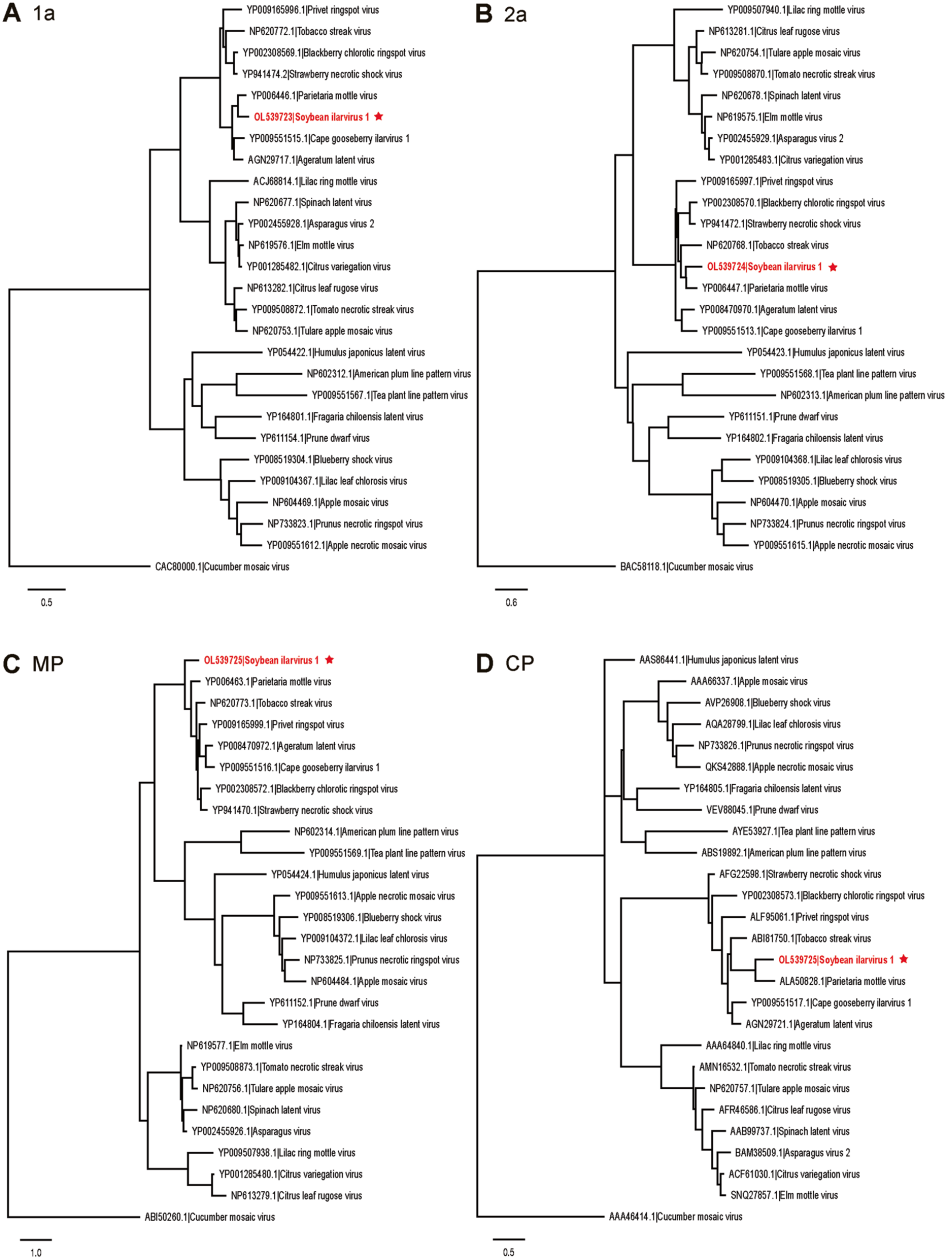
Maximum likelihood phylogenetic tree showing the relationship of the soybean ilarvirus 1 (SIlV1) to other ilarviruses. The phylogenetic tree was based on the amino acid sequences of **A** 1a, **B** 2a, **C** MP, and **D** CP of SIlV1 and other ilarviruses. CMV is included as an outgroup. Sequences were aligned using MUSCLE, and the tree constructed from the alignment using PhyML. The tree branches were bootstrapped with 1000 replications. The de novo assembled SIlV1 genome identified in this study, along with its respective accession number are denoted by a red (*) star

To further investigate the phylogenetic relationship of SIlV1 to 26 other ilarviruses, we performed multiple sequence alignments using the replicase (ORF1a), RdRp (ORF2a), MP, and CP aa sequences and generated phylogenetic trees (Fig. 8). Maximum-likelihood trees based on the aa alignments showed that SIlV1 is a new member of subgroup 1 that includes PMoV, blackberry chlorotic ringspot virus (BCRV), TSV, strawberry necrotic shock virus (SNSV), privet ringspot virus (PRSV), ageratum latent virus (AgLV), and cape gooseberry ilarvirus 1 (CGIV-I) (Fig. 8).

### Occurrence of typical soybean viruses and mixed infections

We detected six plant viruses well known to infect soybean: TSV, AMV, TRSV, SbDV, BPMV, and SVNV, and they occurred in both single and mixed infections (Tables 1, 2, and Additional files 11 and 12: Tables S1 and S2). The presence of all viruses in field samples (S1-S78) was verified by PCR amplification using detection primers designed from the assembled contigs (Additional file 13: Table S3) followed by Sanger sequencing (Additional file 4: Fig. S4).

**Table 2:** Occurrence of mixed viral infections in field-grown soybean samples collected across the USA

Sample Identity (ID)	State	Year Collected	Mixed Viruses Identified	Blast Hits to Isolates Identified in this Study	Nucleotide Identity (%)
S6	Iowa	2017	Alfalfa mosaic virus	IA-2017	99.8
S6	Iowa	2017	Bean pod mottle virus	IA-2-2017	100
S11	Iowa	2017	Tobacco streak virus	IA-3-2017	100
S11	Iowa	2017	Tobacco ringspot virus	IA-2-2017	100
S12	Iowa	2017	Alfalfa mosaic virus	IA-2017	99.8
S12	Iowa	2017	Tobacco ringspot virus	IA-1-2017	100
S13	Iowa	2017	Alfalfa mosaic virus	IA-2017	99.8
S13	Iowa	2017	Tobacco ringspot virus	IA-2-2017	100
S18	Iowa	2018	Alfalfa mosaic virus	IA-4-2018	100
S18	Iowa	2018	Tobacco streak virus	IA-2-2018	99.9
S20	Iowa	2018	Alfalfa mosaic virus	IA-1-2018	99.9
S20	Iowa	2018	Soybean dwarf virus	IA-1-2018	100
S21	Iowa	2018	Tobacco streak virus	IA-2-2018	99.9
S21	Iowa	2018	Soybean dwarf virus	IA-2-2018	100
S24	Iowa	2018	Alfalfa mosaic virus	IA-1-2018	99.9
S24	Iowa	2018	Tobacco streak virus	IA-2-2018	99.9
S25	Iowa	2018	Alfalfa mosaic virus	IA-1-2018	99.9
S25	Iowa	2018	Tobacco streak virus	IA-2-2018	99.9
S26	Iowa	2018	Alfalfa mosaic virus	IA-1-2018	99.9
S26	Iowa	2018	Tobacco streak virus	IA-2-2018	99.9
S31	Indiana	2016	Soybean vein necrosis virus	IN-3-2016	100
S31	Indiana	2016	Tobacco streak virus	WI-2016	97.8
S34	Maryland	2016	Soybean vein necrosis virus	MD-2016	98
S34	Maryland	2016	Tobacco streak virus	MD-2016	100
S37	Alabama	2016	Soybean vein necrosis virus	AL-3-2016	100
S37	Alabama	2016	Tobacco streak virus	WI-2016	97.8
S40	Ohio	2017	Soybean vein necrosis virus	MD-2016	98
S40	Ohio	2017	Alfalfa mosaic virus	OH-2-2017	100

TSV and AMV were each present in 27 of the 78 samples. In BLASTX searches, the TSV sequences from all 27 samples had > 94% amino acid identity to the reference sequences (GenBank accession numbers: RNA1: ACJ38087.1, ALF12288.1; RNA2: AMB72705.1, ALF12289.1, ACJ38088.1; RNA3: ACJ38090.1, ALF12291.1) (Additional file 15: Table S5). TSV was present in samples from Iowa, Wisconsin, Indiana, Maryland, and Alabama. Sequence analysis suggested that 10 distinct isolates were represented in these samples: IA-2016 (S3), IA-1-2017 (S9), IA-2-2017 (S10), IA-3-2017 (S11), IA-1-2018 (S22), IA-2-2018 (S23), IA-1-2019 (S27), IA-2-2019 (S28), MD-2016 (S34), and WI-2016 (S38).

A total of 27 samples collected from Iowa, Ohio, and Missouri contained full-length AMV sequences (Tables 1, 2 and Additional files 11 and 12: Tables S1 and S2). In the BLASTX analysis, AMV sequences identified in all 27 samples had > 97% amino acid identity to the reference sequences (GenBank accession numbers: RNA1: AXP79048.1; RNA2: ADO85716.1, AGV15825.1, CBJ56556.1; RNA3: AAL73140.2, AWK57376.1, AWK57376.1) (Additional file 15: Table S5). AMV was present in samples from Iowa, Ohio, and Missouri. Sequence analysis identified 8 distinct isolates: IA-2017 (S4), IA-1-2018 (S15), IA-2-2018 (S16), IA-3-2018 (S17), IA-4-2018 (S18), OH-2017 (S39), OH-2-2017 (S40), and MO-2017 (S41).

TRSV was identified in 10 samples from 4 Iowa counties, USA during the 2017 growing season (Tables 1, 2 and Additional file 12: Table S2). Sequence analysis identified three distinct isolates: IA-1-2017 (S12), IA-2-2017 (S13), and IA-3-2017 (S14) that shared > 91% sequence identity to the TRSV reference genomes previously reported in California USA, Australia, and South Korea (GenBank accession numbers: RNA1: U50869.1; and RNA2: MH427298.1, KJ556850.1) (Additional file 15: Table S5). The presence of the three distinct TRSV isolates infected in *N. benthamiana* and soybean was verified by PCR amplification using detection primers designed from the assembled contigs (Additional file 13: Table S3) followed by Sanger sequencing (Additional file 9: Fig. S9). SbDV was detected in 4 soybean samples from 1 Iowa county, USA collected over the 2016–2018 growing seasons (Tables 1, 2). Sequence analysis suggested 4 distinct isolates IA-2016 (S2), IA-2017 (S8), IA-1–2018 (S20), and IA-2-2018 (S21) that shared > 97% sequence identity to the SbDV reference genome previously reported in Illinois USA (GenBank accession number: KJ786321.1) (Additional file 15: Table S5). BPMV was detected in 3 samples collected from 2 counties in Iowa, USA during the 2017 and 2018 growing seasons (Tables 1, 2). Sequence analysis suggested 3 distinct isolates IA-2017 (S5), IA-2–2017 (S6), and IA-2018 (S19) that shared > 98% sequence identity to the BPMV reference genomes previously reported in Iowa, USA and Kentucky, USA (GenBank accession numbers: RNA1: GQ996948.1, GQ996951.1; and RNA2: AF394609.1, GQ996949.1) (Additional file 15: Table S5).

Finally, SVNV was detected in 12 samples collected from 5 different states (Indiana, Delaware, Maryland, Alabama, and Ohio) in 2016 and 2017 (Table 2; Additional files 11 and 12: Tables S1 and S2). These samples were specifically collected based on vein necrosis symptoms. Sequence analysis identified 8 distinct isolates: IN-1-2016 (S29), IN-2-2016 (S30), IN-3-2016 (S31), DE-2016 (S32), MD-2016 (S33), AL-1-2016 (S35), AL-2-2016 (S36), and AL-3-2016 (S37) that shared > 96% sequence identity to the SVNV reference genomes previously reported in Arkansas USA (Milan_TN isolate; GenBank accession numbers: L RNA: GU722317.1; M RNA: GU722318.1; S RNA: GU722319.1, respectively) (Additional file 15: Table S5). SVNV recently emerged as a soybean pathogen, and previous studies indicated that there is low genetic diversity in this virus. We were interested in whether there has been a change in the genetic variation of this virus over time. In order to test this, the complete sequences of the nucleocapsid protein (NP) genes of the 8 SVNV isolates identified in this study along with the NP genes of 15 previous isolates [16, 68] were obtained. Pairwise comparison of the NP gene of 8 isolates with the 15 isolates revealed identities of 98 to 99.8% at the nucleotide level and 97 to 100% at the amino acid level (Additional file 17: Table S7). Additionally, the pairwise comparison of the NP gene of the 8 new SVNV isolates with each other revealed that there is 96 – 98% nt sequence identity and 97 – 100% amino acid sequence identity. These results suggest that there continues to be low diversity in NP sequences among SVNV isolates.

Mixed infections of viruses that co-occurred in soybean were observed in 14 samples. The co-infections of AMV and TSV were detected in 4 samples (S18, S24, S25, and S26) collected from 4 different Iowa counties in 2018 (Table 2). In 2017, co-infections of AMV and TRSV, as well as AMV and BPMV, were detected in 2 samples (S12 and S13) and one sample (S6) from Iowa, respectively. Additionally, mixed infections of AMV and SbDV were identified in one sample (S20) from Iowa in 2018. Co-infections of TSV and TRSV were found in one sample (S11) collected in 2017 while TSV and SbDV were detected in one sample (S21) collected in 2018 from Iowa. Mixed infections of SVNV and TSV were identified in 3 samples (S31, S34, and S37) from 3 different states (Indiana, Maryland, and Alabama) in 2016, while co-infections of SVNV and AMV were detected in one sample (S40) from Ohio in 2017. The presence of mixed infection of viruses was verified by PCR amplification using detection primers designed from the assembled contigs (Additional file 13: Table S3) followed by Sanger sequencing (Additional file 10: Fig. S10).

## Discussion

The main goal of this study was to determine the identity of viruses associated with virus-like symptoms observed on soybean plants during scouting in the field. A total of 135 soybean samples were collected from soybean fields in Iowa (2016–2019) and other states including Alabama, Delaware, Indiana, Missouri, and Wisconsin (2016–2018), HTS -based sequencing was performed, and subsequent RT-PCR analyses were conducted on individual samples. Customized bioinformatics workflows and alignment-based sequence similarity searches were used to identify the viruses that occurred in individual and mixed infections in 78 of the samples. Of the remaining 57 samples, 27 samples had de novo contig length less than 1000 bp with few hits to known viruses and other pathogens such as bacteria, oomycetes and fungi. None of these samples had partial genomes. Additionally, 23 samples had no hits to viral genome sequence but uncovered pathogens such as bacteria, oomycetes and fungi while the remaining 7 samples had no pathogens detected. Attempts were made to detect known viruses from some of these samples by RT-PCR but the results were negative. The reason these samples had smaller contigs and negative RT-PCR results were most likely due to the quality of samples during sample collection in the fields. Because misleading interpretations of partial genomes for virus identity are possible in HTS studies [63], a conservative approach of only contigs resulting in complete or near-complete (entire coding sequence(s)) viral genomes are reported here. Because HTS also generated data on the entire biota inhabiting the sampled soybean tissues, other organisms such as bacteria, fungi, arthropods, or oomycetes were identified from these samples using different bioinformatics workflows [41].

Several known RNA viruses (TSV, AMV, TRSV, SbDV, BPMV, SVNV, and ClYVV) were detected and identified in symptomatic soybean samples. Because a systematic sampling strategy was not used, these data should not be interpreted to indicate virus prevalence. However, it is interesting that SMV, which is generally regarded as one of the most common viruses of soybean [69], was not found in any sample. Surprisingly, we discovered another potyvirus, ClYVV, in commercial soybean in two different years, which is significant, because cultivated soybean is not generally considered to be a host. The two ClYVV isolates were detected in fields in two different counties in central and southeastern Iowa, USA in 2016 and 2017, respectively. Sequencing and phylogenetic analysis showed that ClYVV IA-2016 and ClYVV IA-2017 shared 96.5% nt sequence identity and were most closely related to ClYVV I89-1 (Japan) and ClYVV-Gm (South Korea), the latter of which was also found in soybean [24].

Previous reports indicated rare/sporadic occurrence of ClYVV in cultivated soybean in South Korea and more recently in Ohio, US [24, 25]. ClYVV is naturally transmitted by soybean aphids and other aphids in a non-persistent manner [70] and has a wide host range. It was originally isolated from white clover (*Trifolium repens*) and causes severe lethal systemic necrosis in several legumes, including broad bean (*Vicia faba*), common bean (*Phaseolus vulgaris L*.) and pea (*P. sativum*) [23, 70–73]. Because we recovered both ClYVV isolates and identified a susceptible soybean variety, we were able to investigate biological properties, such as seed transmission, pathogenicity, and host range. The apparent lack of seed transmission suggests that the emergence of ClYVV in soybean may be due to the virus being transmitted from host plants in the landscape to soybean genotypes that also happen to be susceptible. Seed transmission within the *Potyviridae* is not uncommon [74], however, we are not aware of reports that ClYVV is seed transmitted in other legumes.

Symptoms of mosaic chlorosis and vein clearing were observed on the systemically-infected leaves of *N. benthamiana*, soybean, and broad bean, and systemic necrosis was observed in broad bean cv. Robin Hood. Based on symptom severity, ClYVV IA-2017 was more virulent than ClYVV IA-2016 in all three plant species. ClYVV IA-2017 and ClYVV IA-2016 share 96.5% nucleotide identity with mismatches dispersed across the genome. However, these isolates share 99% amino acid identity with only 13 non-conservative differences distributed among P1, HC-Pro, P3, VPg, NIb, and CP, which provides a short list of amino acid residues to investigate for roles in virulence.

The commercial soybean variety that was susceptible to the ClYVV isolates was originally identified as the host for ClYVV IA-2016, but the original host variety is not known for ClYVV IA-2017. It is interesting that Williams 82 and the 41 NAM parents representing diverse soybean germplasm were not susceptible to either ClYVV isolate. The lack of susceptibility in Williams 82 and the NAM parents is consistent with the idea that cultivated soybean is generally considered to be a non-host for ClYVV [26], and demonstrates that the two ClYVV isolates we describe here did not gain the ability to infect a broad range of soybean germplasm. It is unclear if emerging viruses such as ClYVV may evolve to become more virulent following a jump to a new host such as soybean [75]. It will be interesting to test if over time the ClYVV isolates may evolve to infect more soybean genotypes, which could potentially threaten soybean production. Another interesting avenue to explore is the genetic and molecular mechanisms enabling these ClYVV isolates to infect the Acre Edge 22R269 variety.

In another unexpected finding, we identified a novel virus provisionally named soybean ilarvirus 1 (SIlV1), a member of *Ilarvirus*, which includes the well-known soybean pathogen, TSV. Phylogenetically, SIlV1 is closely related to PMoV, BCRV, TSV, SNSV, PRSV, AgLV, and CGIV-I, which are all members of ilarvirus subgroup 1. Therefore, SIlV1 is the second ilarvirus from subgroup 1 that infects soybean. Our results leading to the discovery of ClYVV and SIlV1 in soybean demonstrate the value of using HTS-based approaches for viruses, and more broadly, pathogen identification [41].

Other groups have used HTS for identifying viruses in soybean and other crops. For instance, soybean leaf materials collected from 172 plants throughout Korea [42] exhibited virus-like symptoms. This study performed RT-PCR using primers that could detect five viruses SMV, soybean yellow mottle mosaic virus (SYMMV), soybean yellow common mosaic virus (SYCMV), PeMoV, and peanut stunt virus (PSV). Subsequently, this study pooled RNA from the samples according to province, and then performed RNA sequencing. Through RNA sequencing they added five additional viruses: cucumber mosaic virus (CMV), tomato spotted wilt virus (TSWV), bean common mosaic virus (BCMV), bean common mosaic necrosis virus (BCNMV), and Wisteria vein mosaic virus (WVMV). While the other viruses have all been shown to infect soybean, the observation of WVMV was the first report of this virus infecting soybean. Additionally, this study did not identify a novel virus. Results of both RT-PCR and RNA sequencing showed that mixed infections are common, with co-infections being more common than single infections. Furthermore, their results show that the spectrum of viruses infecting soybean are different in the Midwestern US versus South Korea. There are no viruses in common between our two studies. Furthermore, they noted that some viruses, like AMV, ClYVV, and SbDV, were not found in their study even though they have been previously found in South Korea, suggesting that these viruses are not common in South Korea.

Not surprisingly, our results are much more similar to those recently reported [25] with respect to the spectrum of viruses identified. This study conducted a multi-site sampling of 42 counties in Ohio and collected a total of 259 samples in 2011 and 2012. Most of the samples were from plants displaying virus-like symptoms, but also included healthy plants from fields in which no virus-like symptoms were found. This study [25] also used a pooling strategy for sequencing by combining the samples collected in each year. They found that BPMV was by far the most common virus based on the number of sequencing reads and subsequent RT-PCR conducted on individual samples. SVNV, TRSV, and TSV were the next most common viruses found in multiple counties in their study. They also found isolated cases of SMV, AMV, ClYVV, BYMV, and soybean Putnam virus (SPuV) each in single fields indicating they were sporadic and not widely occurring. The SPuV was a novel *Caulimovirus* that the group had previously reported [25], but we did not observe it in any of our samples.

Unlike the recent studies reported [25, 42], we did not pool samples prior to generating libraries for RNA sequencing, but instead we elected to make individual libraries from each sample. This strategy is more expensive and time consuming, but it did lend itself to assembly of the viral genomes present in each sample without the need for resequencing individual samples. This approach also facilitated comparison of individual virus genomes and direct identification of samples containing mixed infections. Since our initial goal was to identify viruses at the single plant level and not conduct a systematic survey, this approach was acceptable despite the higher cost and time involved. However, it is interesting to consider the case of a systematic survey that could involve hundreds to thousands of plant samples. In those cases, a pooling strategy would be necessary.

Similar to the study reported in 2016 [25], we found that TSV was widely distributed, genetically diverse, and it occurs in mixed infections with SVNV. The presence of TSV in a coinfection with SVNV in a sample from Alabama in 2016 also represents a first report of TSV in soybean in this state. Both viruses are transmitted by thrips [18, 76], and so, it may be expected that conditions favoring thrips could result in frequent co-infections by these viruses. It is interesting that the re-emergence of TSV in soybean was previously reported throughout the Midwest including Iowa, Kansas, and Wisconsin as well as Ontario, Canada [76, 77]. The re-emergence of TSV in soybean in Iowa in 2013 occurred in several counties and was associated with irregular, black streaks and necrotic areas on pods and plants that tested positive for TSV using ELISA [76]. TSV was also reported in several counties in Illinois from 2006–2008 [78] as well as in 2013 [76] and in Ohio in 2011 and 2012 [25]. The occurrence and genetic diversity of TSV identified in 2016–2019 demonstrates that it remains a threat to soybean production, and that further work is warranted to investigate sources of inoculum, which may be due to its wide host range [79–81], seed transmission [82], and thrips transmission [83, 84]. Climatic conditions such as hot and dry weather are favored for thrips propagation for TSV transmission. Additionally, high incidences of TSV among weeds bordering agricultural fields could serve as a source of TSV inoculum.

Like TSV, AMV was also genetically diverse and frequently found in our study, being present in 27/78 soybean samples of which 23 samples originated from 7 counties in Iowa during 2017 and 2018 growing seasons, and in samples collected in Ohio and Missouri in 2017. In previous studies, AMV has caused yield loss in soybean when introduced during early vegetative growth stages with final incidence of infected plants exceeding 30% [85]. Currently, there are limited reports on the detection of AMV in field-grown soybean plants [86–88], with fewer reports in the Midwest states that include Wisconsin [85, 89] and Nebraska [90], and no recent reports of AMV detected in Iowa. The occurrences of AMV in 2017 and 2018 could be due to its wide alternate host range [91–93]. Since AMV is found commonly in alfalfa and this host represents a potential source of inoculum, there are possibilities for the movement of AMV into soybean growing in neighboring fields. The combination of seed transmission [94] and the rapid dispersal of soybean aphids (*Aphis glycines* Matsumura) [87, 95] may also be responsible for recurrent detection of AMV in Iowa.

SVNV, a *Tospovirus* transmitted by soybean thrips, recently emerged in the United States. It was originally reported in Arkansas and Tennessee in 2008 [96], and later also first reported in Iowa and Wisconsin from surveys conducted in 2013 [18, 76, 97, 98]. Since its discovery, SVNV has become prevalent in all major soybean growing regions across North America [16, 25] and was found in more than 98% of the soybean fields [15]. The pairwise alignments of SVNV isolates from 8 samples from four different states (Indiana, Delaware, Maryland, and Alabama) suggest that there were at least 8 different SVNV isolates present in 2016. However, analysis of SVNV population structure revealed that there was not significant diversity among the SVNV isolates identified from 3 states (Indiana, Delaware and Alabama) and that the virus populations are not rapidly changing.

TRSV, a *Nepovirus*, was found in 10/78 soybean samples from 4 counties in Iowa during 2017 growing season. The pairwise alignments of TRSV isolates suggest that there were at least 3 different TRSV isolates present in 2017, with the two RNA genomes (RNA1 and RNA2) sharing > 90% nt sequence identities between the IA-1-2017, IA-2-2017, and IA-3-2017 isolates. The remaining 7 TRSV-positive samples had the two RNA genomes sharing > 99% nt sequence identities to IA-3-2017 isolate. TRSV can cause severe disease in soybean. In particular, TRSV-induced bud blight significantly reduces yield and quality in soybeans. TRSV has a wide host range [99] and its primary source of transmission in soybean remains unclear. However, TRSV can be seed transmitted at a low rate [100, 101] and is transmitted by nematodes and inefficiently spread by several insects including thrips and grasshoppers [6].

BPMV and SbDV were found in a few of the samples displaying virus-like symptoms collected in Iowa. BPMV, a *Comovirus*, is generally considered to be widespread in the major soybean-growing areas in the US and has also significantly increased throughout the north central region [25, 102, 103]. These incidences of BPMV have been attributed to increases in bean leaf beetles as a major mode of transmission [102, 104–106]; although other sources of transmission such as seed-to-seedling and alternative leguminous weed hosts have been reported [104]. BPMV has been one of the most prevalent soybean viruses in Iowa for several years [107]. SbDV, a *Luteovirus*, was detected in 4 samples from 1 county during 2016–2018 growing seasons. SbDV was first reported in soybean in the USA in 2003 in Wisconsin and since then detected in several states including Iowa [6]. SbDV has a limited host range [6] with no reports of seed transmission in soybean. However, some SbDV isolates from the USA were reported to be transmitted efficiently from soybean to soybean and clover and from clover to clover and soybean by soybean aphids [108, 109]. This raises the interesting possibility of ClYVV and SbDV moving between clover and soybean by similar mechanisms.

Several mixed infections were identified in samples collected from Iowa: AMV/TSV, AMV/TRSV, AMV/BPMV, AMV/SbDV, TSV/TRSV, and TSV/SbDV. Mixed infections of SVNV and TSV were detected in 3 different states (Indiana, Maryland, and Alabama), while co-infections of SVNV and AMV was also found in Ohio, USA. A recent report identified mixed infections of multiple viruses in field-grown soybean from a multi-site survey in Ohio using HTS [25]. They identified several SVNV-positive samples co-infected with BPMV, TRSV, ClYVV or BYMV, while BPMV-positive samples were also co-infected with TSV, TRSV, or SMV. These observations coupled with the work by Jo et al. [42] in Korea show that mixed infections are quite common wherever soybeans are produced. The occurrence of mixed infections of viruses in soybean can alter disease symptoms, transmission and pathogenicity [110–112]. However, mixed infections may not necessarily cause serious problems on plants [113], either due to the equilibrium maintained among viruses or possibly due to the convergent evolution of viruses toward mild interactions with the host [114]. An interesting line of study in the future may be to develop a better understanding of how and under what conditions the variety of mixed infections occur, and which ones have the highest potential for impact on crop yields.

## Conclusions

HTS-based virus identification was performed to investigate plant viruses associated with virus-like symptoms in soybean fields during the 2016–2019 growing seasons. Most of the samples were positive for a single virus, and co-infections with more than one virus were also common. Most of the viruses identified, namely, TSV, AMV, TRSV, SbDV, BPMV, and SVNV were previously known to infect soybean. Although we did not conduct a systematic study, the frequency and locations of samples in which TSV, AMV, and TRSV were detected suggest that they are relatively widespread in Iowa soybean fields, and that infections by other viruses, such as SbDV and BPMV were more sporadic. In addition, this is the first report of ClYVV discovered in a commercial soybean variety in Iowa, USA, and the first in which such ClYVV isolates were biologically characterized. Because ClYVV has a broad distribution, there are possibilities that this virus could become prevalent in the future given the right combination of susceptible host genotypes and environmental conditions that promote aphid transmission. Therefore, future studies are needed to understand the genetic and molecular factors that underlie the ability of some ClYVV isolates to infect certain soybean genotypes. The discovery of the novel SIlV1 further justifies the need for more systematic and extensive surveys to identify the viruses infecting soybean. Such information could be used to determine the occurrence and geographical distribution of these viruses to develop and establish control and management strategies to avoid further spread. Virus diagnostics in soybean using HTS provides information needed to anticipate possible disease problems, inform breeding programs, and to develop robust diagnostic methods.

## Supplementary Information

**Additional file 1** Figure S1 Representative images of symptomatic soybean tissues collected during the field scouting for metagenomics analysis. (**A**-**D**) Leaves showing necrosis. A-D represent samples S49, S58, S56, and S63 respectively. (**E**, **F**) Leaves with brown spots. E-F represent samples S50 and S59 respectively. (**G**-**I**) Leaves exhibiting puckering. G-I represent samples S51, S74, and S75 respectively. (**J**-**N**) Leaves with yellow spots. J-N represent samples S55, S71, S72, S73, and S76 respectively. (**O**-**V**) Leaves showing chlorosis. O-V represent samples S57, S64, S65, S66, S67, S68, S69, and S70 respectively. (**W**) Yellowing and discolored leaves. W represents sample S61. (**X**) Brown veins on leaves. X represents sample S62. (**Y**) Leaves showing yellow vein chlorosis. Y represents sample S77

**Additional file 2** Figure S2. Workflow for virus detection and discovery in soybean using next generation RNA sequencing. (**A**) Collection of diseased soybean tissues from different fields during four growing seasons, extraction of total RNA, cDNA library preparation, and Illumina RNA sequencing. (**B**) Bioinformatics pipeline used for virus detection and discovery from sequencing reads. The trimmed raw reads were mapped to soybean genomes (nuclear, mitochondrion and chloroplast). The unmapped reads were then mapped to a known virus list as reference, which included 20,433 plant viral sequences. The mapped viral reads and the non-soybean-non-viral reads were de novo assembled, followed by all-organism NCBI nucleotide and protein databases through BLASTN and BLASTX searches using Blastplus v2.7.1

**Additional file 3** Figure S3. Pairwise alignment of the full-length genome sequences of ClYVV IA-2016 and ClYVV IA-2017 identified in this study following RNA sequencing and assembly

**Additional file 4** Figure S4. Plant viruses confirmed by Sanger sequencing in field grown soybean plants. Reverse transcription-polymerase chain reaction (RT-PCR) was performed in 78 field samples. The PCR products were visualized in a 1% agarose gel electrophoresis containing SYBR Safe DNA gel stain. The PCR products were cleaned using ExoSAP-IT PCR product cleanup reagent and sequenced on Sanger sequencing platform. Primer sequences were designed using Primer3 plus from assembled contigs and the primers were used in this study.

**Additional file 5** Figure S5. Symptoms caused by ClYVV-IA-2016 and ClYVV-IA-2017 in N. benthamiana at 10 days post inoculation (dpi). Healthy mock-inoculated controls are indicated. (**A**) Whole plant infected with ClYVV IA-2016. (**B**) Systemic leaf showing symptoms (right). (**C**) Whole plant infected ClYVV IA-2017. (**D**) Systemic leaf showing symptoms (right)

**Additional file 6** Figure S6. ClYVV-IA-2016 and ClYVV-IA-2017 confirmed by Sanger sequencing in green house grown N. benthamiana, soybean, and broad bean infected plants. Reverse transcription-polymerase chain reaction (RT-PCR) was performed in all 18 samples including mock-inoculated controls. The PCR products were visualized in a 1% agarose gel electrophoresis containing SYBR Safe DNA gel stain. The PCR products were cleaned using ExoSAP-IT PCR product cleanup reagent and sequenced on Sanger sequencing platform. Primer sequences were designed using Primer3 plus from assembled contigs and the primers were used in this study

**Additional file 7** Figure S7. Symptoms caused by ClYVV-IA-2016 and ClYVV-IA-2017 in broad bean. Healthy mock-inoculated controls are indicated. Soybean infected with ClYVV-IA-2016 or ClYVV-IA-2017 was used as an inoculum to infect broad bean plants by mechanical inoculation. (A) Whole plants (cv. Robin Hood) infected with ClYVV-IA-2016 and ClYVV-IA-2017 at 21 days post inoculation (dpi). (B) Whole plants (cv. Broad Windsor) infected with ClYVV-IA-2016 and ClYVV-IA-2017 at 21 dpi. (C) RT-PCR detection of ClYVV isolates in broad bean infected plants. Agarose gel electrophoresis of PCR confirming the presence of ClYVV-IA-2016 and ClYVV-IA-2017 identified by RNA sequencing. Lanes 1 and 4: Mock- inoculated controls of Robin Hood and Broad Windsor respectively; 2 and 5: Detection of ClYVV-IA-2016 in Robin Hood and Broad Windsor respectively; 3 and 6: Detection of ClYVV-IA-2017 in Robin Hood and Broad Windsor respectively; M: 1Kb plus molecular weight maker. Sanger sequencing confirmed amplicon identities. Broad bean cyclophilin (CYP2) reference gene was used as internal controls

**Additional file 8** Figure S8. Detection of clover yellow vein virus (ClYVV) in soybean seedlings. Healthy mock-inoculated controls (C) are indicated. Detection of ClYVV in the crude leaf extracts from mock-inoculated control (C) and ClYVV-infected leaves from (**A**) Parent plants (P1-P5) at 84 days post inoculation (dpi). (**B**) Progeny seedlings (Pr1-Pr5) corresponding to each individual parent plant (P1-P5) at 14 dpi. ImmunoStrip assays were performed using Agdia ImmunoStrip for Potyvirus Group assay. Because ClYVV is the only potyvirus present, we interpret a positive test as indicating its presence. The healthy mock-inoculated leaf extract was used as controls. The control and test lines are indicated by arrows on the right. The ClYVV-negative and ClYVV-positive results are indicated by the symbols (−) and (+), respectively

**Additional file 9** Figure S9. Tobacco ringspot virus (TRSV) sequences confirmed by Sanger sequencing in green house grown N. benthamiana, and soybean infected plants following mechanical inoculation. Reverse transcription-polymerase chain reaction (RT-PCR) was performed in all 6 samples including mock-inoculated controls. The PCR products were visualized in a 1% agarose gel electrophoresis containing SYBR Safe DNA gel stain. The PCR products were cleaned using ExoSAP-IT PCR product cleanup reagent and sequenced on Sanger sequencing platform. Primer sequences were designed using Primer3 plus from assembled contigs and subsequently used in this study

**Additional file 10** Figure S10. Mixed infections of viruses confirmed by Sanger sequencing in field-grown soybean plants. Reverse transcription-polymerase chain reaction (RT-PCR) was performed in all samples identified with mixed infections. The PCR products were visualized in a 1% agarose gel electrophoresis containing SYBR Safe DNA gel stain. The PCR products were cleaned using ExoSAP-IT PCR product cleanup reagent and sequenced on Sanger sequencing platform. Primer sequences were designed using Primer3 plus from assembled contigs and subsequently used in this study

**Additional file 11** Table S1. Sample location, year collected, associated symptoms, and viruses identified with their assigned GenBank accession numbers in field-grown soybean across the United States of America

**Additional file 12** Table S2. Sample location and virus sequence identified from field-grown soybean samples collected across the United States of America. The viruses were identified from the all-organism NCBI nucleotide and protein databases through BLASTN and BLASTX searches using Blastplus v2.7.1. The virus identified for each sample had high percent nucleotide identity (> 99%) to the corresponding virus isolates identified in this study

**Additional file 13** Table S3. Primers used in this study

**Additional file 14** Table S4. Summary of sequencing and virus genome assembly results from field-grown soybean samples collected from 2016-2019 across the United States of America

**Additional file 15** Table S5. Virus genome BLAST alignment results from field-grown soybean samples collected from 2016-2019 growing seasons across the United States of America

**Additional file 16** Table S6. Systemic responses of host genotypes to inoculation with soybean mosaic virus-N (N), clover yellow vein virus-No.30 (No.30), HC-Pro derived chimeric N or ClYVV-No.30a

**Additional file 17** Table S7. Percent (A) nucleotide and (B) amino acid sequence identities of nucleocapsid protein (NP) gene of 8 SVNV isolates identified in this study. The NP genes of 8 isolates are IN-1-2016. IN-2-2016, IN-3-2016, DE-2016, MD-2016, AL-1-2016, AL-2-2016, and AL-3-2016. The NP genes of KY29, KY34, KY35, KY43, KY44, KY102, KY105, KY108, KY112, KY113, KY115, KY117, KY124 (GenBank accession numbers KJ955706 to KJ955718), SVNaV-TN isolate (GenBank accession number HQ728387), and Milan_TN isolate (GenBank accession number GU722319.1) were used for comparison with the 8 SVNV isolates identified in this study

## Data Availability

All Illumina RNA-Seq raw data generated during this study (2016, 2017, 2018 and 2019 growing seasons respectively) were deposited at DDBJ/EMBL/GenBank under the BioProject PRJNA730888, BioProject PRJNA730918, and BioProject PRJNA731151. All datasets generated for this study are included in the manuscript and/or the Supplementary files. GenBank accession numbers of full-length or near-full length contigs of viruses are provided in Table 1 and Supplementary Table S1. All other data generated or analyzed in this study are included in this published article and its supplementary files.

## Abbreviations

HTS: High-throughput sequencing
RNA-seq: RNA sequencing
SbDV: Soybean dwarf virus
AMV: Alfalfa mosaic virus
TSV: Tobacco streak virus
SIlV1: Soybean ilarvirus 1
TRSV: Tobacco ringspot virus
BPMV: Bean pod mottle virus
SVNV: Soybean vein necrosis virus
ClYVV: Clover yellow vein virus
SYMMV: Soybean yellow mottle mosaic virus
SMV: Soybean mosaic virus
SYCMV: Soybean yellow common mosaic virus
PSV: Peanut stunt virus
PeMoV: Peanut mottle virus
PMoV: Parietaria mottle virus
BCRV: Blackberry chlorotic ringspot virus
SNSV: Strawberry necrotic shock virus
PRSV: Privet ringspot virus
AgLV: Ageratum latent virus
CGIV-1: Cape gooseberry ilarvirus 1
CMV: Cucumber mosaic virus
TSWV: Tomato spotted wilt virus
BCMV: Bean common mosaic virus
BCNMV: Bean common mosaic necrosis virus
WVMV: Wisteria vein mosaic virus
MP: Movement protein
CP: Coat protein
RdRp: RNA dependent RNA polymerase
