# Supplementary figures and images for: Detection and discovery of plant viruses in soybean by metagenomic sequencing
Source: Virol J. 2022 Sep 13;19:149. doi: 10.1186/s12985-022-01872-5 (PMC9472442; doi:10.1186/s12985-022-01872-5)

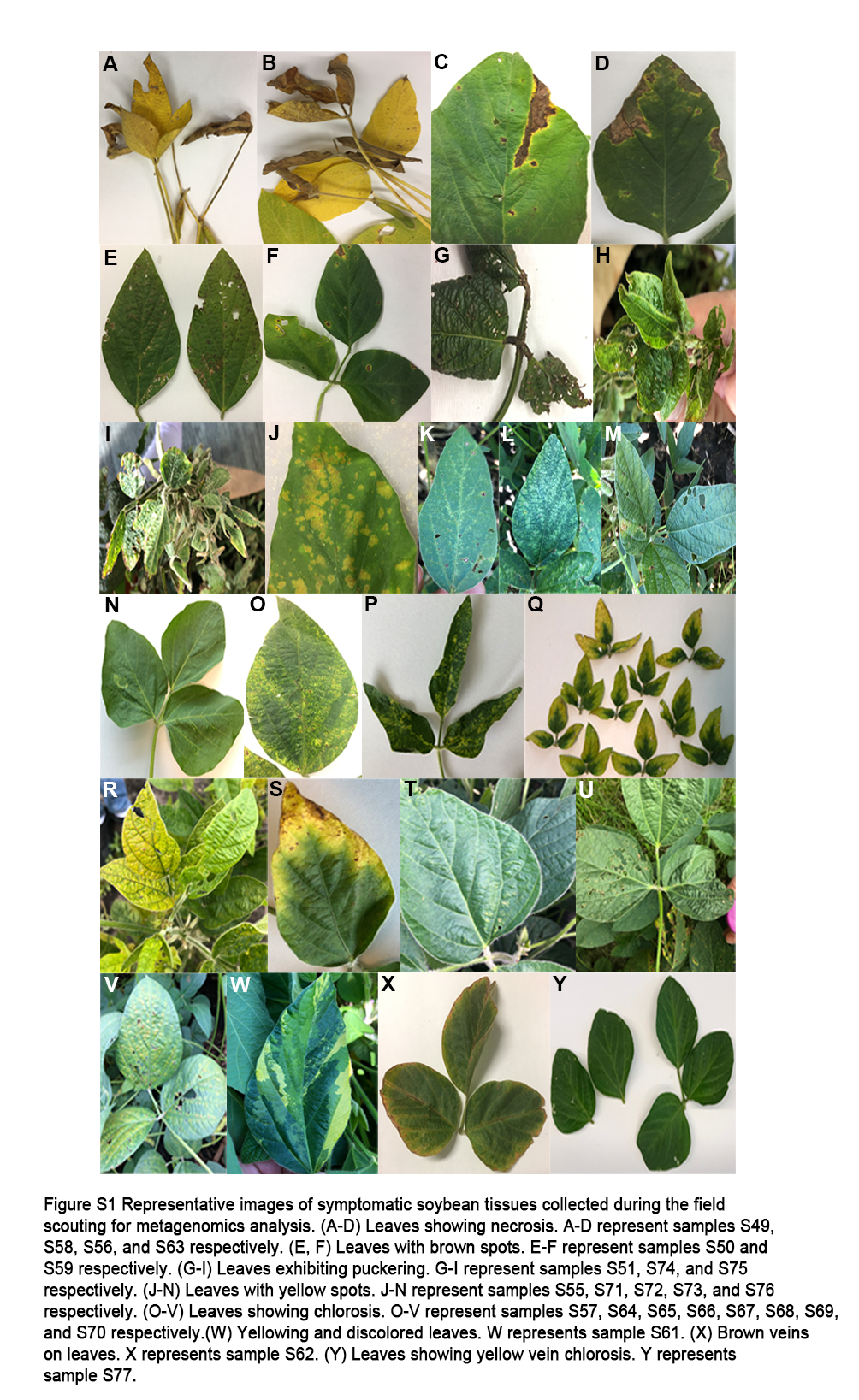

Supplement: Supplementary file 1 — Additional file 1 Figure S1 Representative images of symptomatic soybean tissues collected during the field scouting for metagenomics analysis. (A-D) Leaves showing necrosis. A-D represent samples S49, S58, S56, and S63 respectively. (E, F) Leaves with brown spots. E-F represent samples S50 and S59 respectively. (G-I) Leaves exhibiting puckering. G-I represent samples S51, S74, and S75 respectively. (J-N) Leaves with yellow spots. J-N represent samples S55, S71, S72, S73, and S76 respectively. (O-V) Leaves showing chlorosis. O-V represent samples S57, S64, S65, S66, S67, S68, S69, and S70 respectively. (W) Yellowing and discolored leaves. W represents sample S61. (X) Brown veins on leaves. X represents sample S62. (Y) Leaves showing yellow vein chlorosis. Y represents sample S77 [file 12985_2022_1872_MOESM1_ESM.tif]

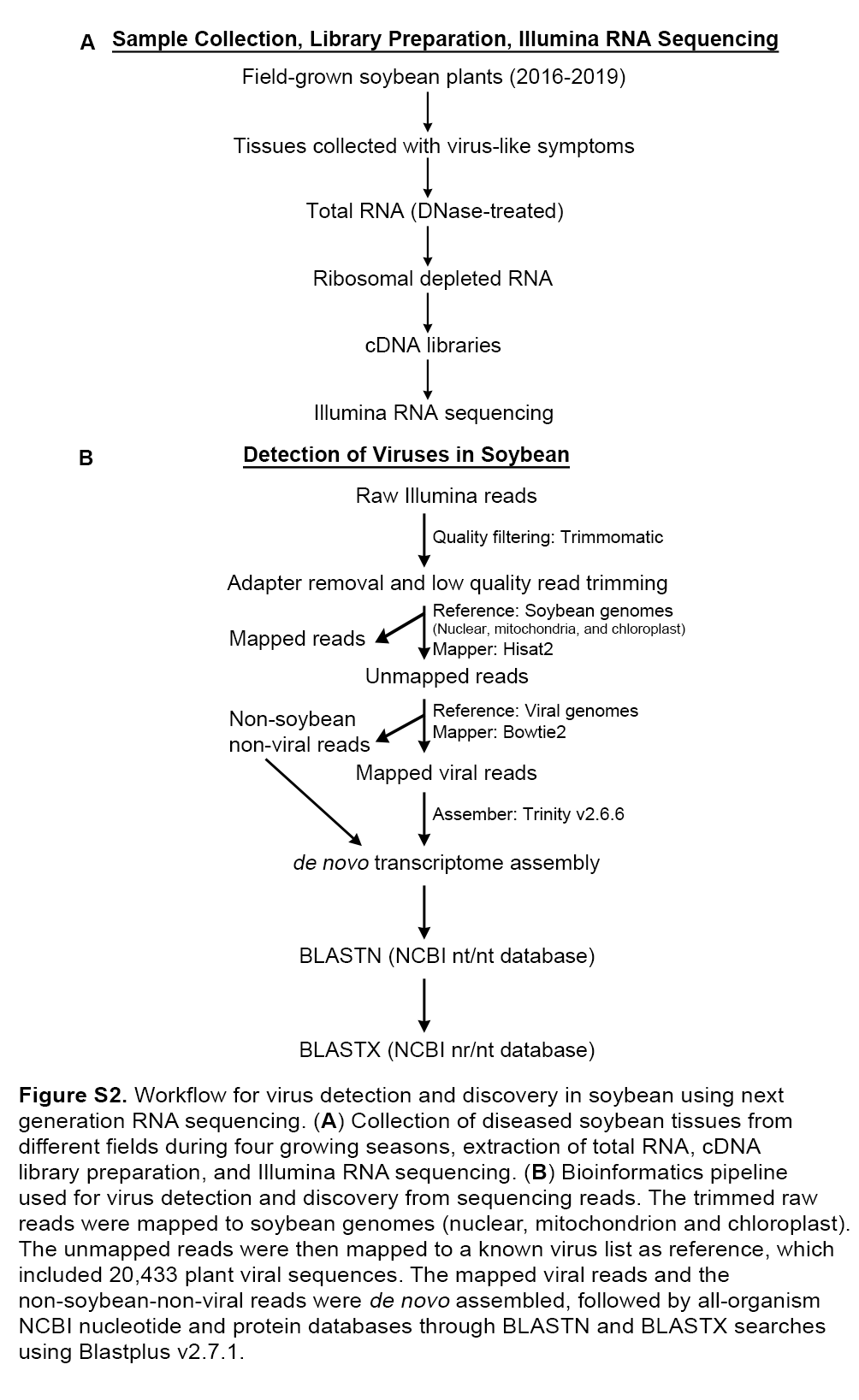

Supplement: Supplementary file 2 — Additional file 2 Figure S2. Workflow for virus detection and discovery in soybean using next generation RNA sequencing. (A) Collection of diseased soybean tissues from different fields during four growing seasons, extraction of total RNA, cDNA library preparation, and Illumina RNA sequencing. (B) Bioinformatics pipeline used for virus detection and discovery from sequencing reads. The trimmed raw reads were mapped to soybean genomes (nuclear, mitochondrion and chloroplast). The unmapped reads were then mapped to a known virus list as reference, which included 20,433 plant viral sequences. The mapped viral reads and the non-soybean-non-viral reads were de novo assembled, followed by all-organism NCBI nucleotide and protein databases through BLASTN and BLASTX searches using Blastplus v2.7.1 [file 12985_2022_1872_MOESM2_ESM.tif]

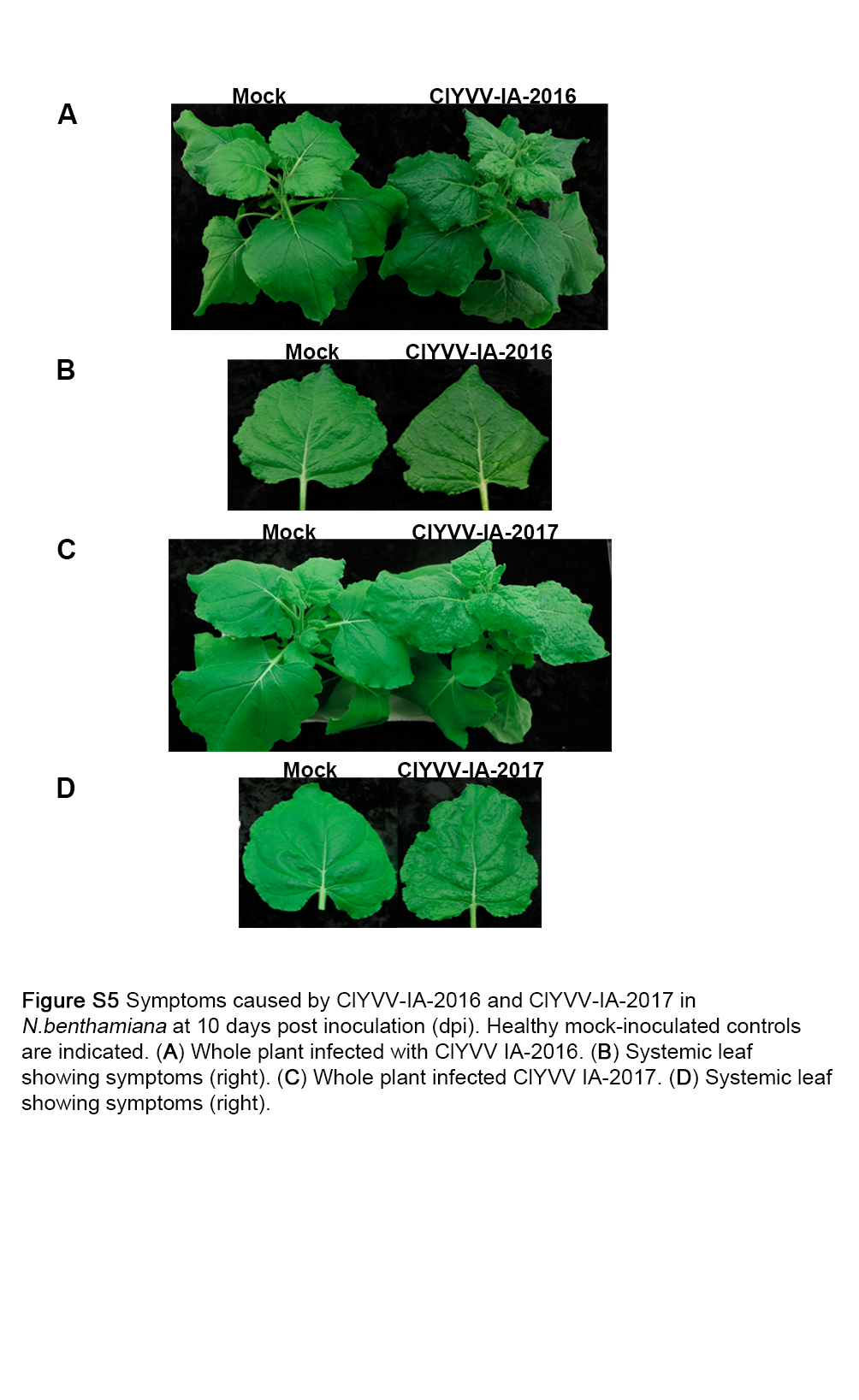

Supplement: Supplementary file 5 — Additional file 5 Figure S5. Symptoms caused by ClYVV-IA-2016 and ClYVV-IA-2017 in N. benthamiana at 10 days post inoculation (dpi). Healthy mock-inoculated controls are indicated. (A) Whole plant infected with ClYVV IA-2016. (B) Systemic leaf showing symptoms (right). (C) Whole plant infected ClYVV IA-2017. (D) Systemic leaf showing symptoms (right) [file 12985_2022_1872_MOESM5_ESM.tif]

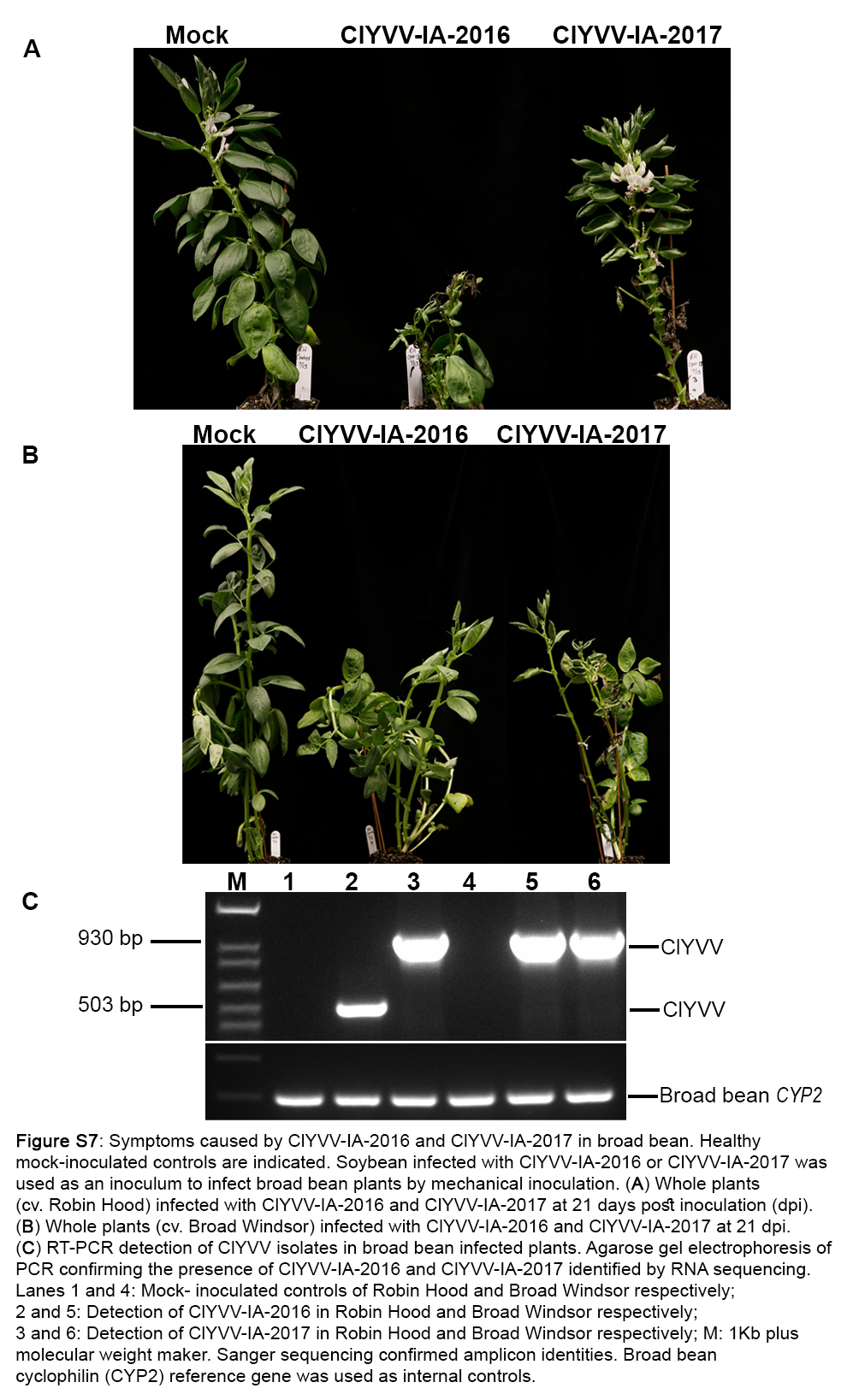

Supplement: Supplementary file 7 — Additional file 7 Figure S7. Symptoms caused by ClYVV-IA-2016 and ClYVV-IA-2017 in broad bean. Healthy mock-inoculated controls are indicated. Soybean infected with ClYVV-IA-2016 or ClYVV-IA-2017 was used as an inoculum to infect broad bean plants by mechanical inoculation. (A) Whole plants (cv. Robin Hood) infected with ClYVV-IA-2016 and ClYVV-IA-2017 at 21 days post inoculation (dpi). (B) Whole plants (cv. Broad Windsor) infected with ClYVV-IA-2016 and ClYVV-IA-2017 at 21 dpi. (C) RT-PCR detection of ClYVV isolates in broad bean infected plants. Agarose gel electrophoresis of PCR confirming the presence of ClYVV-IA-2016 and ClYVV-IA-2017 identified by RNA sequencing. Lanes 1 and 4: Mock- inoculated controls of Robin Hood and Broad Windsor respectively; 2 and 5: Detection of ClYVV-IA-2016 in Robin Hood and Broad Windsor respectively; 3 and 6: Detection of ClYVV-IA-2017 in Robin Hood and Broad Windsor respectively; M: 1Kb plus molecular weight maker. Sanger sequencing confirmed amplicon identities. Broad bean cyclophilin (CYP2) reference gene was used as internal controls [file 12985_2022_1872_MOESM7_ESM.tif]

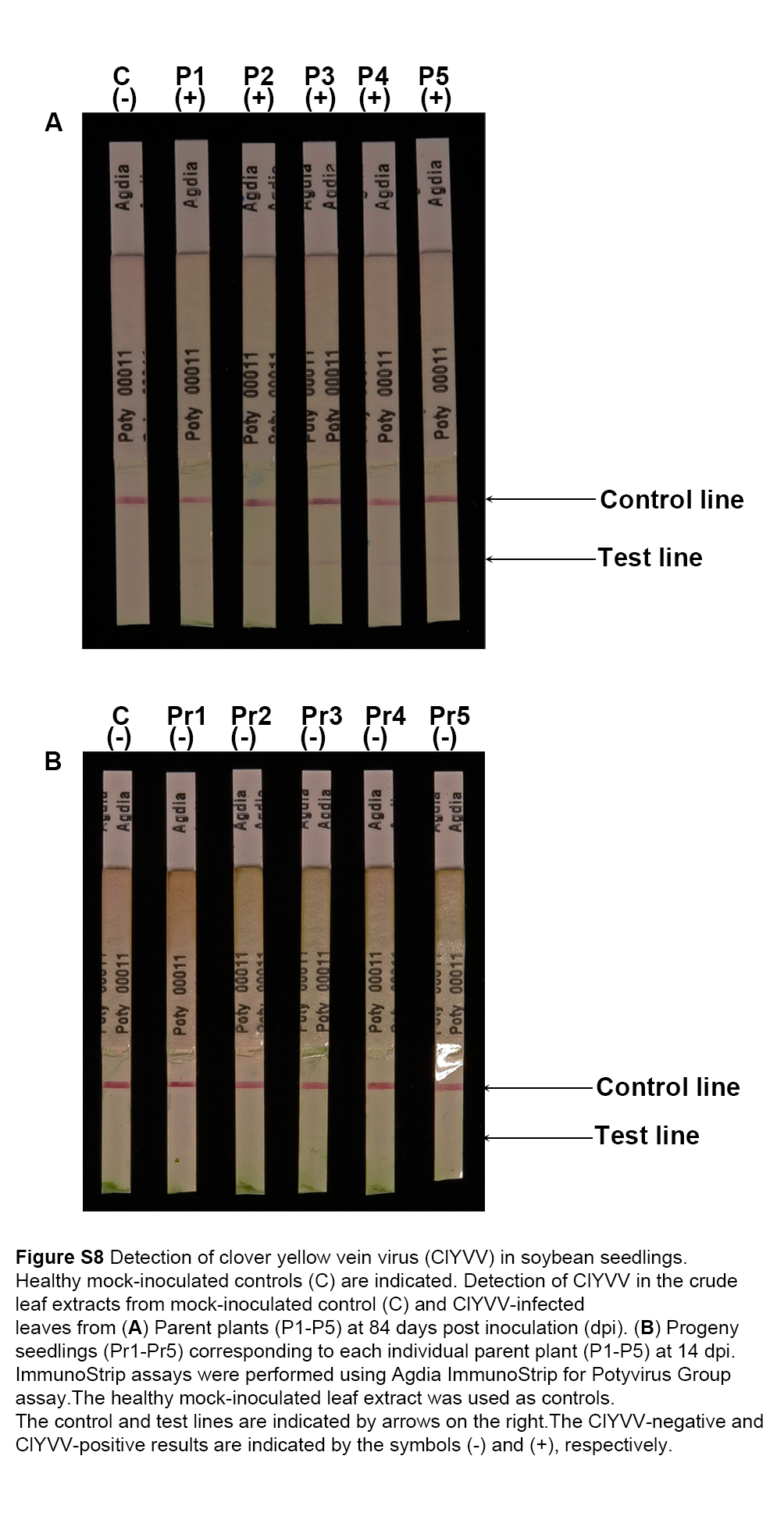

Supplement: Supplementary file 8 — Additional file 8 Figure S8. Detection of clover yellow vein virus (ClYVV) in soybean seedlings. Healthy mock-inoculated controls (C) are indicated. Detection of ClYVV in the crude leaf extracts from mock-inoculated control (C) and ClYVV-infected leaves from (A) Parent plants (P1-P5) at 84 days post inoculation (dpi). (B) Progeny seedlings (Pr1-Pr5) corresponding to each individual parent plant (P1-P5) at 14 dpi. ImmunoStrip assays were performed using Agdia ImmunoStrip for Potyvirus Group assay. Because ClYVV is the only potyvirus present, we interpret a positive test as indicating its presence. The healthy mock-inoculated leaf extract was used as controls. The control and test lines are indicated by arrows on the right. The ClYVV-negative and ClYVV-positive results are indicated by the symbols (−) and (+), respectively [file 12985_2022_1872_MOESM8_ESM.tif]
