# Supplementary material for: Detection and discovery of plant viruses in soybean by metagenomic sequencing
Source: Virol J. 2022 Sep 13;19:149. doi: 10.1186/s12985-022-01872-5 (PMC9472442; doi:10.1186/s12985-022-01872-5)
Supplement: Supplementary file 3 — Additional file 3 Figure S3. Pairwise alignment of the full-length genome sequences of ClYVV IA-2016 and ClYVV IA-2017 identified in this study following RNA sequencing and assembly [file 12985_2022_1872_MOESM3_ESM.pdf]

Figure S3 Pairwise alignment of the full-length genome sequences of ClYVV IA-2016 and ClYVV IA-2017 identified in this study following RNA sequencing and assembly.

```
ClYVV-IA-2016      GATAATCAACTCCAAAATGGGAAAAATATAAAATCAATACAAGACAAATACAGACAAAGC
ClYVV-IA-2017      ------------------------------------------GACAAATACAGACAAAGC
                                                             ******************

ClYVV-IA-2016      AATCAAACTCTCGAATTTTCAAGCATTCAAGCAAGTTCACACAAACGAAGCAAAGTAAGC
ClYVV-IA-2017      AATCAAACTCTCGAATTTTCAAGCATTCAAGCAAGTTCACACAAACGAAGCAAAGTAAGC
                   ************************************************************

ClYVV-IA-2016      CAACAATCTTCTGTAAAATCACTTCAGTTTCGCATTTCTTTAATTTTATTTGCAATCTCT
ClYVV-IA-2017      CAACAATTTTCTGTAAGATCACTTCAGTTTCGCATTTCTTTAATTTTATTTGCAATCTCT
                   ******* ******** *******************************************

ClYVV-IA-2016      TATTTGTAATTTTAGTATAATTTTATATAAGCAATGGCACAAGTTATGATTGGAAGCATC
ClYVV-IA-2017      TATTTGTAATTTTAGTATAATTTTATATAAGCAATGGCACAAATTATGATTGGAAGCATT
                   ****************************************** **************** 

ClYVV-IA-2016      ATGGTTCCCTTAAACAAGGAATCTAAAGTTTCTGAAACTAATATAAGTACTAGTAGTAAT
ClYVV-IA-2017      ATGGTTCCCTTAAACAAGGAATCTAAAGTTTCTGAAACTAATATAAGTACTAGTAGTAAT
                   ************************************************************

ClYVV-IA-2016      ATTCCTAAAACAAAAACCAGCACTCACATCGAGGCTGGACCCAAAAACATGTCTTTTAAC
ClYVV-IA-2017      ATTCCTAAAACAAAAACCAGCACTCACATCGAGGCTGGACCCAAAAACATGTCTTTTAAC
                   ************************************************************

ClYVV-IA-2016      TTACAACCAACCACTGATCCATTCAAAGCGATAACTCTAGCTTGCAAAGAAAGAGTTGCA
ClYVV-IA-2017      TTACAACCAACCACTGATCCATTCAAAGCAATAACTCTAGCTTGCAAAGAAAGAGTTGCA
                   ***************************** ******************************

ClYVV-IA-2016      AAGTTTGGTGACATGGTCTTTAAGACTAGAAGAGGCATCACCACAATGGTGCCAAAACCA
ClYVV-IA-2017      AAGTTTGGTGACATGGTCTTTAAGACTAGAAGAGGCATCACCACAATGGTGCCAAAACCA
                   ************************************************************

ClYVV-IA-2016      GCACGCTTGGTTGAGGAGGAAAAGAGAGCAGCTGAAGCTCTAAAGCGATTCATGGAAGTG
ClYVV-IA-2017      GCACGTTTGGTGGAGGAGGAAAGGAGAGCAGCTGAAGCTCTAAAGCGATTCATGGAAGTG
                   ***** ***** ********** *************************************

ClYVV-IA-2016      GAGTGGACTCTGGAAGAAATTGATCCTGATGCTGAATACCAAGTGCCAGATTTAAGACAG
ClYVV-IA-2017      GAGTGGACTCTGGAAGAAATTGATCCTGACGCTGAATACCAAGTGCCAGACTTAAGACAG
                   ***************************** ******************** *********

ClYVV-IA-2016      AGGGGTTCCCAAGTGTCATTTAAGAGTCATTACCACAAGAGGACTCCAAAAATAGCAAAG
ClYVV-IA-2017      AGGGGTTCCCAAGTGTCATTTAAAAGTCCTTACCACAAGAAGACTCCAAAAATAGCAAGG
                   *********************** **** *********** ***************** *

ClYVV-IA-2016      AAATTTAAAGTGGTTAAGCCAGTGCAAGGGAACAAGAAAATTCAGCATGTAACAAATGCA
ClYVV-IA-2017      AAACTTAAAGTGGTTAAGCCAGTGCAAGGGAACAAGAAAATTCGGCATGTAACAAATGCA
                   *** **************************************** ***************

ClYVV-IA-2016      CTGCTCAAGATTGTTAAGAATAACAATCTAGTTCTTGAAGTTGCTGACAGAAATAAGAAA
ClYVV-IA-2017      CTGCTCAAGATTGTTAAAAATAACAATCTAGTCCTTGAAGTTGCTGACAGGAATAAGAAA
                   ***************** ************** ***************** *********

ClYVV-IA-2016      GCAAATCATGCAACTTTCTCAAAATATGGCTCAACTTATGGCATGCACATAATTGTTAAC
ClYVV-IA-2017      GCAAATCACGCAACTTTCTCAAAATATGGCTCAACTTATGGCATGCACATAATTGTTAAC
                   ******** ***************************************************

ClYVV-IA-2016      CATATGGTTCGAAAGAGACGCTCAGTTGATGTGCGACTTAATGGTCTCATGACAAGCATT
ClYVV-IA-2017      CATATGGTTCGAAAGAGACGTTCAGTTGATGTGCGACTCAATGGTCTCATGACAAGCATT
                   ******************** ***************** *********************
```

```
ClYVV-IA-2016      GCAAAGCAGGAAGCAATTGGATTTGAAAAGTTGAATGTTAGCACATTAAGAGAGGGGCAC
ClYVV-IA-2017      GCAAAGCAGGAAGCAATTGGATTTGAAAAGTTGAATGTTAGCACATTAAGAGAGGGGCAC
                   ************************************************************

ClYVV-IA-2016      AGTGGACTTGTGTTGCAAACCAAAACAGTCCCAAACTGCCACTTCATTAATGATGACATA
ClYVV-IA-2017      AGTGGACTAGTGTTGCAAACCGAAACAGTCCCAAACTGCCACTTCAATAATGATGACATA
                   ******** ************ *********************** *************

ClYVV-IA-2016      ACAATTGTGAGAGGTGTAATAAAGAGTCATGGAATACCTTGCTTAGTAGATGCTAGACAG
ClYVV-IA-2017      ACAATTGTGAGAGGTGTGATAAAGAGTCATGGAATACCTTGCTTAGTAGATGCTAGACAG
                   ***************** ******************************************

ClYVV-IA-2016      AATTTGAATCATCAGCAACTTAGTAGGATAAGGGAATTCTCTGCAGGAGATCTGTTTTGG
ClYVV-IA-2017      AGTTTGAATTATCAACAACTGAGTAGGATAAGGGAATTCTCTGCAGGAGATCTATTTTGG
                   * ******* **** ***** ******************************** ******

ClYVV-IA-2016      AAAGGATACAATCAGACTTTCATAGATAATAGACCAAAAGACATTGGACACACATGTACA
ClYVV-IA-2017      AAAGGATACAATCAGACTTTTATAGATAATAGACCAAAAGACATTGGACACACATGTACA
                   ******************** ***************************************

ClYVV-IA-2016      TCTGATCTAAACGTAGTCCAATGTGGTTCCGTGATGGCGTTAATGACTCTGGCACTATTC
ClYVV-IA-2017      TCTGATCTTAACGTAGTCCAATGTGGTTCCGTGATGGCGTTGATGACTCTGGCACTATTC
                   ******** ******************************** ******************

ClYVV-IA-2016      CCGTGTGGAAGAATCACTTGCAAAGAATGTGTGGAGGATTTTCATAATCAGAACAATAGA
ClYVV-IA-2017      CCGTGTGGAAGAATCACCTGCAAAGAATGTGTGGAGGATTTTCATAATCAGAACAATAGG
                   ***************** *****************************************

ClYVV-IA-2016      GAGAGATATGCAAGAACAGAAAGATTCTTAAGCCAGGCAATGACTCTCCTAGCACAAACA
ClYVV-IA-2017      GAGAGATATGCAAGAACAGAAAGATTCTTAAGCCAGGCAATGACTCTCCTAGCACAAACA
                   ************************************************************

ClYVV-IA-2016      TACCCAGAATTCAAACACTCAGGAGACATCTTGCAATTATTTCGTGAGCGTTTGAGTATG
ClYVV-IA-2017      TACCCAGAATTCAAACACTCAAGAGACATCTTGCAATTATTTCGTGAGCGTTTGAGTATG
                   ********************* **************************************

ClYVV-IA-2016      GAAAACTCAAATGCAGAGGCCTGTGTTGAGACAAACAAAGCCATAACATCACTTACAGAG
ClYVV-IA-2017      GAAAACTCAAATGCAGAGGCCTGTGTTGAGACAAACAAAGCCATAACATCACTTACAGAG
                   ************************************************************

ClYVV-IA-2016      ACTCCATTTAATCACATAAGGAAGATAAATGAGGTGCTTATAAGATATGGTAGTTTAACA
ClYVV-IA-2017      ACTCCATTTAATCACATAAGGAAGATAAACGAGGTGTTTATAAGATATGGTAGTTTAACA
                   ***************************** ****** ***********************

ClYVV-IA-2016      AATGATGAAGTGGGGGTTGCAAGTGCGAGCTTACTTGAAATTACTAGGTACATTCGAAAC
ClYVV-IA-2017      AATGATGAAGTGGGGGTTGCAAGTGCGAGCTTACTTGAAATTACTAGGTACATTCGAAAT
                   ***********************************************************

ClYVV-IA-2016      AGAACTGATTCCATCCAAAGGAATGACCTAAGTAAGTTCAGAAACAAGATTTCTTCAAAG
ClYVV-IA-2017      AGAACTGATTCCATCCAAAGGAATGACCTAAGTAAGTTCAGAAACAAGATTTCCTCAAAG
                   ***************************************************** ******

ClYVV-IA-2016      ACACACATCAACTTAGATTTGATGTGTGATAACCAACTTGACAAAAATGCAAACTTCATG
ClYVV-IA-2017      ACACACATCAACTTAGATTTGATGTGTGATAACCAACTTGACAAAAATGCAAACTTCATG
                   ************************************************************

ClYVV-IA-2016      TGGGGTCAGAGAGCATACCATGCAAAACGATTTTTGTCGAATTATTTCAGTGTGATTGAT
ClYVV-IA-2017      TGGGGTCAGAGAGCATATCATGCGAAACGATTTTTGTCGAATTATTTCAGTGTGATTGAT
                   ***************** ***** ************************************

ClYVV-IA-2016      CCATCTGAAGGTTACGATAAATTCATCAAGAGAAAGCTACCCAATGGTACAAGAGAACTT
ClYVV-IA-2017      CCATCTGAAGGTTATGACAAATTCATCAAGAGAAAGCTACCCAATGGTACAAGAGAACTT
                   ************** ** ******************************************
```

```
ClYVV-IA-2016      GCAACTGGTAGGTTGATCGTTCCTACAAACTTTGAATCTTTTAGAGATCAGATGAAAGGA
ClYVV-IA-2017      GCAACTGGCAGGTTGATCGTTCCTACAAACTTTGAATCTTTTAGAGATCAAATGAAAGGA
                   ******** ***************************************** *********

ClYVV-IA-2016      ACCATGGTGGACAATAGACCAATTGGCAAGGAATGTGTTTCGCGTGTAAAAGGAGCCTTT
ClYVV-IA-2017      ACCATGGTGGACAATAGACCAATTGGCAAGGAATGTGTTTCGCGTGTTAAAGGAGCCTTT
                   *********************************************** ************

ClYVV-IA-2016      TGTTACCCGTGCTGTTGCACAACTGATGATATTGGCAATGCAATTTTGTCTGAATTTAAA
ClYVV-IA-2017      TGTTACCCGTGCTGTTGCACAACTGATGATATTGGCAATGCAATTTTGTCTGAGTTTAAA
                   ***************************************************** ******

ClYVV-IA-2016      ATGCCAACAAAATATCATTTAGTTCTTGGTGGTCAGGAAGCAGCGAAATACATAGAGTTA
ClYVV-IA-2017      ATGCCAACAAAATATCATTTAGTTCTTGGTGGCCAGGAAGCAGCGAAATACATAGAGTTA
                   ******************************** ***************************

ClYVV-IA-2016      CCATCAGATAGCAGTGGTATGATGTACATAGCGAAAGACGGATATTGCCATATCAATATA
ClYVV-IA-2017      CCATCAGATAGCAGTGGCATGATGTACATAGCAAAAGATGGATATTGCCATATCAATATA
                   ***************** ************** ***** *********************

ClYVV-IA-2016      TTCTTTGCTATGTTGGTGAATGTGTCAGAAGATAAATCAAAGGATTTCACCAAAATGGTG
ClYVV-IA-2017      TTCTTTGCTATGTTGGTGAATGTGTCAGAAGATAAATCAAAGGACTTCACTAAAATGGTG
                   ******************************************** ***** *********

ClYVV-IA-2016      CGGGATCAGATAATGCCGAAGCTAGGTCAGTGGCCAACGATGCTAGATGTTGCAACAGCG
ClYVV-IA-2017      CGGGATCAGATAATGCCGAAGCTAGGTCAGTGGCCAACAATGCTAGATGTTGCAACAGCA
                   ************************************** ********************

ClYVV-IA-2016      TGTTGGTACTTAACAGTTTGGTTTCCTGATACACTTAGTGCTGAACTTCCGAGAATACTT
ClYVV-IA-2017      TGTTGGTACTTAACAGTTTGGTTTCCTGATACACTTAGTGCTGAACTTCCAAGAATACTT
                   ************************************************** *********

ClYVV-IA-2016      GTGGATCACAAGCTAAGCACCATGCATGTTCTAGATTCATATGGTTCTATAAGCACAGGA
ClYVV-IA-2017      GTGGATCACAAGCTTAACACCATGCATGTTCTAGATTCATATGGTTCGATAAGCACAGGA
                   ************** * ****************************** ************

ClYVV-IA-2016      TATCATGTTCTCAAGGCAAATATTGTTTCCCAATTAATCAAGTTTGCTAGTGATGATCTT
ClYVV-IA-2017      TATCATGTTCTCAAGGCAAATATTGTTTCTCAATTGATCAAGTTTGCTAGTGATGATCTT
                   ***************************** ***** ************************

ClYVV-IA-2016      GAGTCAGATTTGAAGTTTTACAGAGTTGGTGGCAAATCATTGACAGGGCAGGTGATACAG
ClYVV-IA-2017      GAGTCAGATTTGAAATTCTACAGAGTTGGTGGCAAATCATTAACAGGGCAAGTGATACAG
                   ************** ** *********************** ******** *********

ClYVV-IA-2016      TTTGACACAAAAATGTTAATCTCAAGTATTTACCGGCCAAGACAGATGGAGAGGATCATC
ClYVV-IA-2017      TTTGACACAAAAATGTTAATCTCAAGTATCTACCGACCAAGACAGATGGAGAGGATCATC
                   ***************************** ***** ************************

ClYVV-IA-2016      AATGAAGAACCTTTTGTGCTAGTTCTAGCAATGCAATCACCATCAGTTCTTTTAGCCTTG
ClYVV-IA-2017      AATGAAGAGCCCTTTGTGCTGGTTCTAGCAATGCAATCACCATCAGTTCTTTTAGCCTTG
                   ******** ** ******** ***************************************

ClYVV-IA-2016      TTCAATAGTGCTTCGCTAGAGAAAGCCGTGGAGGTCTGGCTACACAAGGACATGCGCGTT
ClYVV-IA-2017      TTCAATAGTGCCTCGCTAGAGAAAGCCGTGGAGGTTTGGCTGCACAAGGACATGCGCGTT
                   *********** *********************** ***** ******************

ClYVV-IA-2016      TCACATGTGATGACAATGCTTGCCCTCCTGGCAGCAAAAGTAAGTGCGGCCAAGATGGTG
ClYVV-IA-2017      TCACATGTGATGACAATGCTTGCCCTCCTGGCAGCAAAAGTAAGTGCAGCCAAGATGGTG
                   *********************************************** ************
```

```
ClYVV-IA-2016      AATTTACAGATGGAGATAATAGAAGCTAGCGCTAGCCACTTTCTTGCTGCAATGGACACC
ClYVV-IA-2017      AATTTACAGATGGAGATAATAGAAGCTAGTGCTAGCCACTTTCTCGCTGCAATGGACACC
                   ***************************** ************** ***************

ClYVV-IA-2016      ATTCACAAGCCAATGCATTCCATTAACACAGCAAACATTTTCTTGATGAACTTAGAAGAA
ClYVV-IA-2017      ATTCACAAGCCAATGCATTCCATCAACACAGCGAATATTTTCTTGATGAACTTAGAAGAA
                   *********************** ******** ** ************************

ClYVV-IA-2016      GGGAGATCAACTGATAGAACAATTGATGAACTTGGTTTTCATTCTTTGAAAAAGTCTAGC
ClYVV-IA-2017      GGGAGATCAACTGACAGAACAATTGACGAACTTGGGTTCCACTCTTTGAAAAAGTCTAGC
                   ************** *********** ******** ** ** ******************

ClYVV-IA-2016      CAGGTACTCATGGAAAAAATCTGGGCAGAGGATTTAGAGCAGCAATGGCTAGGTTTAAGA
ClYVV-IA-2017      CAAGTACTCATGGAAAAAATCTGGGCAGAGGATTTAGAGCAGCAATGGCTAGGTTTAAGA
                   ** *********************************************************

ClYVV-IA-2016      TTGTCACAAAAGTTTTATTTAATAAGGCAGTCATGGAAGCAGCGGGCAAAGTATTCCAAA
ClYVV-IA-2017      TTGTCGCAAAAGTTTTATTTAATAAGGCAGTCATGGAGGCAGCGGGCAAAATATTCCAAA
                   ***** ******************************* ************ *********

ClYVV-IA-2016      ATATTAGCCCAGAGAGACGAGCTAGGTGCCAGCGACAAATTCAGCGCATCACTCAGATTG
ClYVV-IA-2017      ATACTAGCCCAGAGAGACGAGCTAGGTGTCAGCGACAAGTTCAGCGCATCACTCAGATTG
                   *** ************************ ********* *********************

ClYVV-IA-2016      TCAGCAACAAGCATCAAAAATCAAGCAATCAGTTGCAGGAAGAGAATGGTCGCAACAAGT
ClYVV-IA-2017      TCAGTAACAAGCGTCAAGAATCAAGCAATCAGTTGTAGGAAGAGAATGGTCGCAACAAGT
                   **** ******* **** ***************** ************************

ClYVV-IA-2016      AAGAAGTGTTTGTTCAGTGTGCAGAAAATGGTAGCAATGCAAGCCTTAAGGGTGTTCAAA
ClYVV-IA-2017      AAGAAGTGTTTGTTCAGTGTGCAGAGAATGGTAGCAATGCAAGCATTAAAGGTGTTTAAA
                   ************************* ****************** **** ****** ***

ClYVV-IA-2016      CGATGCATGAGTAATATGGTGGATGTTCTCAACGTGTTAGCAACAATAACACTCCTGATG
ClYVV-IA-2017      CGATGCATGAGAAATATGGTGGATGTTCTCAACGTGTTAGCAGCAATAACACTCCTGATG
                   *********** ****************************** *****************

ClYVV-IA-2016      GGTATCCTGAGTCAAGTGAGATCCCACATCAAAACTGTCACATCGTACAAGCGCGTTTCA
ClYVV-IA-2017      GGCATCCTGAGCCAAGTGAGATCCCACATCAAAACTGTTACATCGTACAAGCGCGTTTCA
                   ** ******** ************************** *********************

ClYVV-IA-2016      AAGGAAGCAAAAGTTCAAGATGACTTGTACAGAATCAATGAGTATTACGAGTTGTTGAAA
ClYVV-IA-2017      AAGGAAGCAAAAGTTCAAGATGACTTGTACAGAATCAATGAGTATTATGAGTTGTTGAAA
                   *********************************************** ************

ClYVV-IA-2016      GCTCGTGATAGGTACACAGTGGATGAGTTTCGCAGTAAACTGGAATCACTAAACCCAGAA
ClYVV-IA-2017      GCTCGTGATAGGTATACAGTAGATGAGTTCCGCAGTAAGCTGGAATCATTGAACCCAGAA
                   ************** ***** ******** ******** ********* * *********

ClYVV-IA-2016      CTCCTAGAGACTTTTGATGAGTACTACAAGGAACCAAAGTGGTTTGTCATGGAGTCCAAG
ClYVV-IA-2017      CTCCTAGAGACTTTTGATGAGTACTACAAGGAACCAAAGTGGTTTGTCATGGAGTCAAAG
                   ******************************************************** ***

ClYVV-IA-2016      TCAAATGATATGGTAGCTCTTGAGAAAGTAGTGGCTTTTACAGCACTAGTCATGATGATA
ClYVV-IA-2017      TCAAATGACATGGTTGCTCTTGAGAAAGTAGTGGCTTTTACAGCGCTAGTCATGATGATA
                   ******** ***** ***************************** ***************

ClYVV-IA-2016      TTTGATGGAGAGAGGAGTGATTGCGTCTACAAAATACTGAACAAGCTCAAAGGTATAATC
ClYVV-IA-2017      TTTGATGGAGAGAGGAGTGATTGTGTCTACAAAATACTGAACAAACTTAAAGGTATAATC
                   *********************** ******************** ** ************

ClYVV-IA-2016      TCAACAACAACTCAGGATGGCTACAAATTTCAAAGTCTTGATGACATTAAACCCATGTTG
ClYVV-IA-2017      TCAACAACAACTCAAGATGGTTACAAATTTCAGAGTCTTGATGACATTAAACCCATGTTG
                   ************** ***** *********** ***************************
```

```
ClYVV-IA-2016       GAAGATAAAAAGGGTGTTATTGATTTTGAGATTGATGAGGGTGACAGCAAGATAATATGC
ClYVV-IA-2017       GAAGACAAGAAGGGTGTTATTGATTTTGAGATTGATGAGGGTGATAGCAAGATAATATGC
                    ***** ** *********************************** ***************

ClYVV-IA-2016       AGCAACCAGACAACATTCGTTCAGTGGTGGGATAATCAACTCCAAAATGGGAATGTCATC
ClYVV-IA-2017       AGCAACCAAACAACGTTCGTTCAGTGGTGGGATAATCAACTTCAAAATGGGAATGTCATT
                    ******** ***** ************************** *****************

ClYVV-IA-2016       ACACACTATAGAACAGAAGGCCATTTCATGGAATTCACCAGAGAGACAGCACAAGAGGTT
ClYVV-IA-2017       ACACACTATAGAACAGAAGGCCATTTCATGGAATTCACCAGAGAGACAGCACAAGAAGTT
                    ******************************************************** ***

ClYVV-IA-2016       GCGCATACGATAGCCCACAGCGATTTTAAAGACATCTTAGTTAGAGGGGCTGTTGGATCG
ClYVV-IA-2017       GCACATACGATAGCCCACAGTGATTTTAAAGACATCTTAGTGAGAGGGGCTGTTGGATCG
                    ** ***************** ******************** ******************

ClYVV-IA-2016       GGAAAATCAACTGGTTTACCATCATATCTTAGTGAGAAGGGGAAGGTGTTGATGCTGGAG
ClYVV-IA-2017       GGAAAATCAACTGGTTTACCATCATATCTTAGTGAGAAGGGGAAGGTGTTGATGCTGGAA
                    ***********************************************************

ClYVV-IA-2016       TCAACAAGACCATTAGCAGAAAATGTATTCAAGCAGCTCAAGAGCGATCCCTTCTACAAA
ClYVV-IA-2017       TCAACAAGACCATTAGCAGAAAATGTATTCAAGCAGCTCAAGAGTGATCCCTTCTACAAA
                    ******************************************** ***************

ClYVV-IA-2016       AACCCAACACTCCGCATGAGAGGGACAACGAGTTATGGTTCAACACCTATTACGATCATG
ClYVV-IA-2017       AACCCGACACTTCGCATGAGAGGAACAACGAGTTACGGCTCAACACCTATCACGATCATG
                    ***** ***** *********** *********** ** *********** *********

ClYVV-IA-2016       ACAAGTGGTTTTGCGCTCCATTTTTATGCAAACAATCCCCAGTTACTGAAGGAGTTTCAA
ClYVV-IA-2017       ACAAGTGGTTTTGCACTTCATTTCTATGCAAACAATCCTCAGTTACTGAAGGAATTCCAA
                    ************** ** ***** ************** ************** ** ***

ClYVV-IA-2016       TTCATTATCATAGATGAGTGTCATGTTCTGGATGCCAATGCGATTGCATTTGTCAGCCTG
ClYVV-IA-2017       TTCATTATCATAGATGAGTGCCATGTTCTGGATGCTAATGCAATTGCATTTGTCAGCCTG
                    ******************** ************** ***** ******************

ClYVV-IA-2016       CTCAAGGAGTTCTCATTTCAGGGGAAACTTGTCAAAGCATCAGCAACACCACCAGGGAGA
ClYVV-IA-2017       CTCAAGGAATTTTCATTTCAGGGGAAACTTGTCAAAGCATCAGCAACACCACCAGGGAGA
                    ******** ** ************************************************

ClYVV-IA-2016       GAAACTGAATTCACAACACAACACAAAGTGTCATTGGTTACGCAAGACCAACTTTCTTTT
ClYVV-IA-2017       GAAACTGAATTCACAACACAACACAAAGTGTCATTAGTCACGCAAGACCAACTTTCATTT
                    *********************************** ** ***************** ***

ClYVV-IA-2016       GACCAATTTGTGGCCCAGCAAGGTACAGGTTCCAATTGCGACATGATTGATGTGTGCGAC
ClYVV-IA-2017       GATCAATTTGTGGCCCAGCAAGGTACAGGTTCTAATTGCGACATGATTGATGTGTGTGAC
                    ** ***************************** *********************** ***

ClYVV-IA-2016       AATATACTGGTTTATGTCGCGAGTTACAATGAAGTGGACCAACTCTCAAAGATGTTACTG
ClYVV-IA-2017       AATATACTGGTTTATGTCGCGAGTTACAATGAAGTGGACCAACTCTCAAAGATGTTACTG
                    ************************************************************

ClYVV-IA-2016       GAGAAGGGATTTCTGGTTACGAAAGTTGATGGAAGAACCATGAAAGGAGGAAAGACTGAG
ClYVV-IA-2017       GAAAAAGGATTTCTGGTTACGAAAGTTGATGGAAGAACCATGAAAGGAGGAAAAACTGAG
                    ** ** *********************************************** ******

ClYVV-IA-2016       ATTGAGACGAGGGGAACAAAAGCAAGGAAGCATTTCGTTGTCGCAACAAACATCATAGAG
ClYVV-IA-2017       ATTGAGACGAGGGGAACAAAAGCAAAGAAGCATTTCGTTGTCGCAACAAACATCATAGAG
                    ************************ ***********************************

ClYVV-IA-2016       AATGGAGTGACACTTGACATAGAGGGTGTTGTAGACTTTGGGTTAAAGGTCGTTCCTGAG
ClYVV-IA-2017       AATGGAGTGACACTTGATATAGAGGGTGTTGTAGACTTTGGGTTAAAGGTCGTTCCTGAG
                    ***************** ******************************************
```

```
ClYVV-IA-2016      TTAGACGCGGATAGCAGAGTGATGCGCTATTCGAAGCACAGAGTCTCATTTGGTGAGAGG
ClYVV-IA-2017      TTGGACGCGGATAGCAGAGTGATGCGCTATTCGAAGCACAGAGTCTCATTTGGTGAGAGG
                   ** *********************************************************

ClYVV-IA-2016      ATTCAGAGAATCGGAAGAGTTGGTAGGCACAAAGCTGGTAAAGCACTCAAAATTGGAACA
ClYVV-IA-2017      ATTCAGAGAATCGGAAGAGTTGGTAGGCACAAAGCTGGTAAAGCACTCAAAATTGGAACA
                   ************************************************************

ClYVV-IA-2016      ACAGAGAGGAACCTTCAAAAGATCCCAGAGTTGGTTGCTACAGAGGCAGCATTCTACTGC
ClYVV-IA-2017      ACAGAGAGGAACCTTCAAAAGATCCCAGAGTTGGTTGCTACAGAGGCAGCATTCTACTGC
                   ************************************************************

ClYVV-IA-2016      TTTGCGTACGGACTTCCAGTTATGTCTGAGGGGGTCTCATCCAACATGCTGAGTAAATGC
ClYVV-IA-2017      TTTGCGTACGGACTTCCAGTTATGTCTGAGGGAGTCTCATCCAACATGCTGAGTAAATGC
                   ******************************** ***************************

ClYVV-IA-2016      ACAGTGCCACAAGCAAAGACCATGATGAGTTTTGAGCTACCAATCATGTACACAGTTAAC
ClYVV-IA-2017      ACAGTGCCGCAAGCAAAGACCATGATGAGCTTTGAGTTACCAATCATGTACACAGTTAAC
                   ******** ******************** ****** ***********************

ClYVV-IA-2016      TTGGTGAGGTTTGACGGTTCAATGCATCCATCCATACACGAGTTGCTTAAACCATACAAG
ClYVV-IA-2017      TTGGTGAGGTTTGACGGTTCAATGCATCCATCCATACATGAGTTGCTTAAACCATACAAG
                   ************************************** *********************

ClYVV-IA-2016      CTGAGGGACTCGAATGTTGTTCTAAACAAAATGGCTATACCACATGGGAATGTCAAGAAC
ClYVV-IA-2017      CTGAGGGACTCGAATGTTGTTCTAAACAAAATGGCTATACCACATGGGAATGTCAAGAAC
                   ************************************************************

ClYVV-IA-2016      TGGATTTCGGTCAGAGAGCTCAAGTATGTTGGTGTCAGAATTGACATTCAAGAGGACATC
ClYVV-IA-2017      TGGATTTCAGTCAGAGAGCTCAAGTATGTTGGTGTCAGAATTGACATTCAAGAAGACATC
                   ******** ******************************************** ******

ClYVV-IA-2016      CGGGTACCTTTCCATGCCAGAGACATACCGGACAAGTTACATGAAAGAATCTTTGAGGCC
ClYVV-IA-2017      CGGGTACCCTTCCATGCCAGAGACATACCGGACAAGTTACATGAAAGAATTTTTGAGGCC
                   ******** ***************************************** *********

ClYVV-IA-2016      TGCTTGAAACACAAGGGAGATGCAGGTTTTGGCAGACTAAGCGTAGTTAGCGCATGTAAG
ClYVV-IA-2017      TGCTTGAAACACAAGGGAGATGCAGGTTTTGGCAGACTAAGCGTAGTTAGTGCGTGTAAG
                   ************************************************** ** ******

ClYVV-IA-2016      GTGGCTTATACTTTGCAAACTGACCCTACATCATTACAAAGAACAATCAAGATTCTGGAT
ClYVV-IA-2017      GTGGCTTATACTTTGCAAACTGATCCTACATCACTACAAAGAACAATCAAGATTCTGGAT
                   *********************** ********* **************************

ClYVV-IA-2016      GAGTTGATAGCAAGAGAACAAAGAAAGAGAGAGTACTTCCAGAGTGCTTCAAACACAGCT
ClYVV-IA-2017      GAGTTGATAGCAAGAGAACAAAGAAAGAGAGAGTACTTCCAGAGTGCTTCAAACACAGCT
                   ************************************************************

ClYVV-IA-2016      TGCTCTGGTTCCAGTTACTCCTTGACAAGCATCATCAATGCTATTAAGGCACGTAACACT
ClYVV-IA-2017      TGCTCTGGTTCAAGTTACTCCTTGACAAGCATCATCAATGCTATTAAGGCACGTAACACT
                   *********** ************************************************

ClYVV-IA-2016      TGTGATTTCACTCAAGAGAACTTAAGCGTGCTTCATAGTGCCAGGCAGCAATTGATGGAA
ClYVV-IA-2017      TGTGATTTCACTCAAGAGAACTTAAGCGTACTTCATAGTGCTAGGCAGCAATTGATGGAG
                   ***************************** *********** ***************** 

ClYVV-IA-2016      TTCAAGAACATAAATTGTGATTTTTCAAAACCATCAACGCTTGATGAATTTGGTGCACTG
ClYVV-IA-2017      TTCAAAAACATAAATTGTGATTTTTCAAGACCATCAACGCTTGATGAATTTGGTGCACTG
                   ***** ********************** *******************************

ClYVV-IA-2016      GATTGTTTGCAGTTTGAATCAGCGAGTGAGATAAGCAAGCATTTAAAGCTCAAGAATCAC
ClYVV-IA-2017      GATTGTTTGCAGTTCGAGTCAGCGAGTGAGATAAGCAAGCATTTAAAGCTCAAGAATCAC
                   ************** ** ******************************************
```

```
ClYVV-IA-2016      TGGAATGGATCACTGTTGGCCAAAGATATGCTCATAGCTTTAGGCGTTCTTGGTGGCGGT
ClYVV-IA-2017      TGGAATGGATCACTGTTGGCCAAAGATATGCTCATAGCTTTAGGCGTTCTTGGTGGCGGT
                   ************************************************************

ClYVV-IA-2016      TGTTGGATGTTGTATACTTATTACAACCAGGAAGTGTCCAAAGAATATAAGTTCCAAGGG
ClYVV-IA-2017      TGTTGGATGTTATATACTTACTACAACCAGGAAGTGTCCAAAGAATATAAGTTCCAAGGG
                   *********** ******** ***************************************

ClYVV-IA-2016      AAAAGCAAGAGAACAAGACAAAAGTTGAAGTTCAGAGCGGCGAGAGACATGAAGGATCGT
ClYVV-IA-2017      AAAAGTAAGAGAACAAGACAAAAGTTGAAGTTCAGAGCGGCAAGAGACATGAAGGATCGT
                   ***** *********************************** ******************

ClYVV-IA-2016      TATGAAGTGCATGCCGATGAGGGGACTTTAGTGGAGAATTTTGGAACTCGTTATTCAAAG
ClYVV-IA-2017      TATGAAGTGCATGCCGATGAGGGAACTTTAGTGGAAAATTTTGGAACTCGTTATTCAAAG
                   *********************** *********** ************************

ClYVV-IA-2016      AAAGGCAAGACAAAAGGCACTGTTGTGGGTTTGGGTGCAAAAACAAGACGGTTTACTAAC
ClYVV-IA-2017      AAAGGCAAGACAAAAGGCACTGTTGTGGGTTTGGGTGCAAAAACAAGACGGTTCACTAAC
                   ***************************************************** ******

ClYVV-IA-2016      ATGTATGGTTTTGACCCCACGGAGTATTCATTTGCTAGGTATCTTGATCCAATCACGGGT
ClYVV-IA-2017      ATGTATGGTTTTGACCCCACGGAGTACTCATTTGCTAGGTATCTTGATCCAATTACGGGA
                   ************************** ************************** ***** 

ClYVV-IA-2016      GCAACATTGGATGAAACCCCAATTCACAATGTAAATTTGGTTGCTGAGCATTTTGGCGAC
ClYVV-IA-2017      GCAACATTGGATGAAACTCCAATCCACAATGTAAATTTGGTTGCTGAGCATTTTGGTGAC
                   ***************** ***** ******************************** ***

ClYVV-IA-2016      ATAAGGCTTGACATGGTTGACAGGGAGTTACTTGACAAACAGCACTTATACCTCAAGAGA
ClYVV-IA-2017      ATAAGGCTTGATATGGTTGACAAGGAGTTACTTGACAAACAGCACTTATACCTCAAGAGA
                   *********** ********** *************************************

ClYVV-IA-2016      CCAATAGAATGTTACTTTGTAAAGGATGCTGGTCAGAAGGTGATGAGGATTGATCTAACA
ClYVV-IA-2017      CCAATAGAATGTTACTTTGTTAAGGATGCCGGTCAGAAGGTGATGAGGATTGATCTGACA
                   ******************** ******** ************************** ***

ClYVV-IA-2016      CCCCACAACCCATTGTTGGCAAGCGATGTTAGCACAACCATAATGGGTTATCCTGAGAGA
ClYVV-IA-2017      CCCCACAACCCATTGTTGGCAAGCGATGTTAGCACAACCATAATGGGTTATCCTGAGAGA
                   ************************************************************

ClYVV-IA-2016      GAAGGTGAACTCCGTCAAACTGGAAAGGCAAGGTTAGTCGACCCATCAGAGTTGCCCGTG
ClYVV-IA-2017      GAAGGTGAACTCCGTCAAACTGGAAAGGCAAGGTTAGTCGACCCATCAGAGTTGCCCGCG
                   ********************************************************** *

ClYVV-IA-2016      CGAAATGAGGATATTGATGCAGAGTTTGAAAGTCTAAATCGCATAAGCGGTTTGCGCGAC
ClYVV-IA-2017      CGGAATGAGGATATTGATGCAGAGTTTGAGAGTCTAAATCGCATAAGTGGTTTGCGCGAC
                   ** ************************** ***************** ************

ClYVV-IA-2016      TATAATCCCATTTCACAAAATGTTTGCTTGCTAACAAATGAGTCAGAAGGCCATAGAGAG
ClYVV-IA-2017      TATAATCCCATTTCACAAAATGTTTGCTTGCTAACAAATGAGTCAGAAGGCCATAGAGAG
                   ************************************************************

ClYVV-IA-2016      AAGATGTTTGGAATTGGGTATGGTTCGGTGATCATTACAAATCAACATCTGTTCAGAAGG
ClYVV-IA-2017      AAGATGTTTGGAATTGGATATGGTTCGGTGATCATTACAAATCAGCATCTGTTCAGAAGG
                   ***************** ************************** ***************

ClYVV-IA-2016      AATAATGGGGAGTTGTCAATTCAATCCAAGCATGGCTACTTCAGATGCCGCAACACCACA
ClYVV-IA-2017      AATAATGGGGAGTTATCAATTCAATCCAAGCATGGCTACTTCAGATGCCGCAACACCACA
                   ************** *********************************************

ClYVV-IA-2016      AGCTTGAAGATGCTACCTTTGGAGGGACACGACATTTTGTTAATTCAGTTACCAAGGGAT
ClYVV-IA-2017      AGCTTGAAGATGCTGCCTTTGGAGGGACATGACATTTTGTTGATTCAGTTACCAAGGGAC
                   ************** ************** *********** ***************** 
```

```
ClYVV-IA-2016      TTCCCAGTGTTTCCACAAAAGATTCGCTTCAGGGAGCCAAGAGTGGATGACAAAATTGTT
ClYVV-IA-2017      TTCCCAGTGTTTCCACAAAAGATTCGCTTTAGGGAGCCAAGAGTGGATGACAAAATAGTT
                   ***************************** ************************** ***

ClYVV-IA-2016      TTGGTCAGCACAAATTTCCAGGAAAAGAGCTCCTCGAGCACGGTCTCAGAGTCCAGTAAC
ClYVV-IA-2017      TTGGTCAGCACAAATTTCCAGGAAAAGAGTTCCTCGAGCACGGTCTCAGAGTCCAGCAAC
                   ***************************** ************************** ***

ClYVV-IA-2016      ATTTCAAGAGTGCAGTCAGCCAATTTCTACAAGCATTGGATCTCAACAGTGGCAGGACAC
ClYVV-IA-2017      ATTTCAAGAGTGCAGTCAGCTAATTTCTACAAGCACTGGATCTCAACAGTGGCAGGACAC
                   ******************** ************** ************************

ClYVV-IA-2016      TGTGGAAACCCTATGGTTTCGACTAAAGATGGATTTATTGTAGGTATCCACAGTCTTGCT
ClYVV-IA-2017      TGTGGAAACCCTATGGTTTCAACTAAAGATGGATTTATTGTAGGTATCCACAGTCTTGCT
                   ******************** ***************************************

ClYVV-IA-2016      TCATTGACAGGTGACGTTAACATCTTCACAAGCTTTCCGCCGCAGTTTGAAAACAAATAT
ClYVV-IA-2017      TCATTGACCGGCGATGTTAACATCTTCACAAGTTTTCCGCCGCAGTTTGAGAACAAATAT
                   ******** ** ** ***************** ***************** *********

ClYVV-IA-2016      CTACAGAAGCTCAGTGAACACACATGGTGTAGTGGATGGAAACTAAATCTTGGAAAGATT
ClYVV-IA-2017      CTACAGAAGCTCAGTGAACATACATGGTGTAGTGGATGGAAATTAAATCTTGGAAAGATT
                   ******************** ********************* *****************

ClYVV-IA-2016      AGTTGGGGTGGAATCAACATTGTGGAGGATGCACCTGAAGAGCCCTTTATTACATCCAAG
ClYVV-IA-2017      AGTTGGGGTGGAATCAACATTGTGGAGGATGCACCTGAAGAGCCCTTTATAACATCCAAG
                   ************************************************** *********

ClYVV-IA-2016      ATGGCAAGCCTTCTTAGTGATTTGAATTGTTCATTCCAAGCAAGTGCGCAAACCAAGTGG
ClYVV-IA-2017      ATGGCAAGCCTTCTTAGTGATTTGAATTGTTCATTCCAAGCAAGTGCGCAAACCAAGTGG
                   ************************************************************

ClYVV-IA-2016      TTGTTGAAGGAAGTGAAGGATAATGTGCAGGCAGTAGCTCAGGCCCCAAGTGCTTTAGTT
ClYVV-IA-2017      TTGTTAAAGGAAGTGAAGGATAATGTGCAGGCAGTAGCTCAGGCTCCGAGTGCTTTAGTT
                   ***** ************************************** ** ************

ClYVV-IA-2016      ACAAAACATGTAGTGAAAGGAAAGTGCGCATTGTTTGAAGTGTACTTAGCTAATAATCAG
ClYVV-IA-2017      ACAAAACATGTAGTGAAAGGAAAGTGCGCATTGTTTGAAGTGTACTTAGCCAATAACCAG
                   ************************************************** ***** ***

ClYVV-IA-2016      GAGGCAGAGAAATTCTTTAGACCTATGATGGGCTTCTATCAGAAGAGTAGATTAAACAAA
ClYVV-IA-2017      GAGGCAGAAAAATTCTTTAGACCTATGATGGGCTTCTATCAGAAGAGTAGATTGAACAAA
                   ******** ******************************************** ******

ClYVV-IA-2016      GAAGCTTATACAAAGGATCTCATGAAATACTCAAAGGTGATTGATGTTGGCTTGGTTGAC
ClYVV-IA-2017      GAAGCTTATATAAAGGATCTCATGAAATACTCGAAGGTGATTGATGTTGGCCTGGTTGAC
                   ********** ********************* ****************** ********

ClYVV-IA-2016      ACTGAGAAGTTCGAAAAGGGTTTAACTAAAGTTGAGTGCATGCTAAGACAAAAAGGGTTC
ClYVV-IA-2017      ACTGAGAAGTTCGAAAAGTGTTTAACTAAAGTTGAGTGCATGCTAAGACAAAAAGGGTTC
                   ****************** *****************************************

ClYVV-IA-2016      ACAAATTGCAATTACGTGAACGACGAAGCAGACATCTATGCAGCACTAAATATGAAAGCA
ClYVV-IA-2017      ACAAATTGCAATTACGTGAACGATGAAGCAGACATCTATGCAGCACTAAATATGAAAGCA
                   *********************** ************************************

ClYVV-IA-2016      GCTATGGGAGCTTTGTACTCAGGGAAAAAGAAAGATCATTTCGAAGGCATGCCAATGGAA
ClYVV-IA-2017      GCCATGGGAGCTTTGTACTCAGGGAAAAAGAAAGATCATTTCGAAGGTATGCCAATGGAA
                   ** ******************************************** ************

ClYVV-IA-2016      GAATTTGCCAAATTCATTAGAGCAAGTTGTGAGCGTCTGTTCAGTGGGAAAATGGGAGTT
ClYVV-IA-2017      GAATTTGCCAAATTCATTAGAGCAAGTTGTGAGCGTCTGTTCAGTGGGAAAATGGGAGTT
                   ************************************************************
```

```
ClYVV-IA-2016      TGGAATGGCTCGTTAAAGGCTGAACTTAGACCACAAGAAAAGGTTTTGGCAAACAAGACA
ClYVV-IA-2017      TGGAATGGCTCTTTAAAGGCTGAACTTAGACCACAAGAAAAGGTTTTGGCAAACAAGACA
                   *********** ************************************************

ClYVV-IA-2016      AGGTCCTTCACAGCAGCACCAGTGGACACATTACTGGCTGGAAAGGTTTGTGTGGATGAT
ClYVV-IA-2017      AGGTCCTTCACAGCAGCGCCAGTGGACACATTACTGGCTGGGAAGGTTTGTGTGGACGAT
                   ***************** *********************** ************** ***

ClYVV-IA-2016      TTCAATAACAAGTTCTACAGTCTCCATCTTGAGATACCCTCCACAGTTGGTATCACCAAA
ClYVV-IA-2017      TTCAATAACAAATTCTACAGTCTCCATCTTGAGATACCATCCACAGTTGGTATCACCAAA
                   *********** ************************** *********************

ClYVV-IA-2016      TTTTATGGTGGTTGGGATTCATTGTTGAATAAGCTGCCAGATGGATGGGTGTATTGTGAT
ClYVV-IA-2017      TTTTATGGTGGTTGGGATTCATTGTTGAATAAGCTGCCAGATGGATGGGTGTATTGTGAT
                   ************************************************************

ClYVV-IA-2016      GCTGATGGCTCGCAGTTTGATAGTTCTCTAACACCATACCTGATAAATTCTGTTCTGAGA
ClYVV-IA-2017      GCTGATGGCTCGCAGTTTGATAGTTCTCTAACACCATACCTGATAAATTCTGTTCTGAGA
                   ************************************************************

ClYVV-IA-2016      CTTCGACTGAACTTCATGGAAGAGTGGGATATTGGTGAGGAAATGCTTAAGAACTTGTAC
ClYVV-IA-2017      CTTCGACTGAACTTCATGGAAGAGTGGGACATTGGTGAGGAGATGCTTAGGAACTTGTAC
                   ***************************** *********** ******* **********

ClYVV-IA-2016      ACAGAGATCATATATACACCAATCCTAACACCTGATGGTACAATAATCAAGAAGTTCAAA
ClYVV-IA-2017      ACAGAGATCATATATACACCAATTCTGACACCTGATGGTACAATAATCAAGAAGTTCAAA
                   *********************** ** *********************************

ClYVV-IA-2016      GGAAACAACAGTGGACAACCCTCGACAGTTGTCGATAACACACTAATGGTGATAATGGCT
ClYVV-IA-2017      GGAAACAACAGTGGACAACCCTCGACAGTTGTCGATAACACACTAATGGTGATAATGGCT
                   ************************************************************

ClYVV-IA-2016      ATGTACTATGCAGCAGAAAAACTTGGTGTGGAAGGAGATTTAAGCGAGTCCATTGTGTTC
ClYVV-IA-2017      ATGTACTATGCAGCAGAAAAACTTGGTGTGGAAGGAGATTTAAGCGAGTCCATCGTGTTC
                   ***************************************************** ******

ClYVV-IA-2016      TACGCCAATGGTGATGATTTGTTGATAGCAGTGCACCCAAGTCATGAGTGGTATCTAGAC
ClYVV-IA-2017      TACGCCAATGGTGATGATTTGTTGATAGCAGTGCACCCAAGTCATGAGTGGTATCTAGAC
                   ************************************************************

ClYVV-IA-2016      CAACTTTCTACACTTTTCAAAGAGTTGGGTTTGAACTATGATTTCTCAAGCAGGACAAAG
ClYVV-IA-2017      CAACTTTCTACACTTTTCAAAGAGTTGGGCTTGAACTATGATTTCTCAAGCAGAACAAAG
                   ***************************** *********************** ******

ClYVV-IA-2016      AATAAAGGAGATCTCTGGTTCATGTCACACTGTGGTATAAAGAAAGAGGGTTTGTGGATT
ClYVV-IA-2017      AATAAAGGTGATCTCTGGTTCATGTCACACTGTGGTATAAAGAAAGAAGGTTTATGGATT
                   ******** ************************************** ***** ******

ClYVV-IA-2016      CCCAAGTTGGAGCCAGAAAGAGTAGTCTCAATCCTTGAATGGGATAGAGCGGCAGAGCCT
ClYVV-IA-2017      CCCAAGTTGGAGCCAGAAAGAGTAGTCTCAATCCTTGAATGGGATAGAGCGGCAGAGCCT
                   ************************************************************

ClYVV-IA-2016      GAACATAGGTTGGAAGCTATTTGTGCATCCATGATCGAAGCTTGGGGTTATGATGACTTG
ClYVV-IA-2017      GAACATAGGTTGGAAGCTATTTGTGCATCCATGATCGAAGCTTGGGGTTATGATGACTTG
                   ************************************************************

ClYVV-IA-2016      CTCAATCACATACGCAGGTTTTATTTATGGGTTCTTGACCAAGCACCATACAAACAGCTC
ClYVV-IA-2017      CTCAATCACATACGCAGGTTTTATTTATGGGTTCTTGACCAAGCACCATACAAGCAGCTC
                   ***************************************************** ******

ClYVV-IA-2016      AGCGCAGAAGGAAAAGCACCCTATATCTCAGAAGTTGCGTTGAAGAGTTTGTACACTGGG
ClYVV-IA-2017      AGCGCAGAAGGAAAAGCACCCTATATCTCAGAAGTTGCTTTGAAGAGTTTGTACACTGGG
                   ************************************** *********************
```

```
ClYVV-IA-2016      AAACCAGCTACAAGTTGTGAACTAGAAGTGTACAACAAGATCCATCAAGAGCAGCATGAT
ClYVV-IA-2017      AAACCAGCTACAAGTTGTGAACTAGAAGTTTACAATAAGATCCATCAAGAGCAACACGAT
                   ***************************** ***** ***************** ** ***

ClYVV-IA-2016      GAGTTTGATGACAGCCAGATGAAATTTGTTTTCCAATCCGACAAAGAAAAGTTGAATGCT
ClYVV-IA-2017      GAGTTTGATGACAGCCAGATGAAATTTGTTTTCCAATCCGACAAAGAGAAGTTGAATGCT
                   *********************************************** ************

ClYVV-IA-2016      GGTGAGCAACAAAAGTCAAAGGATAAGGAATCAAGACAAAGAGATCAAGAGGGTGAAAAC
ClYVV-IA-2017      GGTGAGCAACAAAAGTCAAAGGATAAGGAATCAAGACAAAGAGATCAAGAGGGTGAAAAC
                   ************************************************************

ClYVV-IA-2016      TCAAACAGACAAATCATTCCAGACAGAGACATCAATGCAGGCACCACTGGGACTTTTTCA
ClYVV-IA-2017      TCGAACAGACAAATCATTCCAGACAGAGACATCAATGCAGGAACCACTGGGACTTTTTCA
                   ** ************************************** ******************

ClYVV-IA-2016      GTACCCAAATTGAAGAAAATATCAGGAAAGCTCTCATTGCCCAAAATCAAAGGAAAAGGA
ClYVV-IA-2017      GTACCCAAATTGAAGAAAATATCAGGAAAGCCCTCATTGCCCAAAATCAAAGGAAAGGGA
                   ******************************* ************************ ***

ClYVV-IA-2016      CTGCTGAACTTGGACCATTTGTTAGTTTATGTTCCAAACCAAGACGACATCTCTAACAAC
ClYVV-IA-2017      CTGCTAAATCTGGACCATTTGTTAGTTTATGTTCCAAACCAAGATGACATCTCTAACAAC
                   ***** **  ********************************** ***************

ClYVV-IA-2016      ATAGCAACTCAAGAGCAGCTGGAAGCGTGGCATGAAGGAGTCAAGAATGCGTATGAAGTG
ClYVV-IA-2017      ATAGCAACTCAAGAGCAGCTAGAAGCGTGGCATGAAGGAGTTAAGAATGCGTATGAGGTG
                   ******************** ******************** ************** ***

ClYVV-IA-2016      GATGATCAACAAATGGAAATCATATGCAATGGATTGATGGTGTGGTGCATAGAAAATGGC
ClYVV-IA-2017      GATGATCAGCAAATGGAAATCATATGCAATGGATTGATGGTGTGGTGCATAGAGAATGGC
                   ******** ******************************************** ******

ClYVV-IA-2016      ACGTCAGGTGATCTTCAAGGTGAGTGGACAATGATGGATGGAGACAAACAGGTAACATTC
ClYVV-IA-2017      ACGTCAGGTGATCTTCAAGGTGAGTGGACAATGATGGATGGAGAGAAACAGGTAACATTC
                   ******************************************** ***************

ClYVV-IA-2016      CCACTTAAGCCAATTCTTGACTTTGCCAAACCAACATTGAGGCAGATTATGGCCCACTTT
ClYVV-IA-2017      CCACTTAAGCCAATTCTTGACTTTGCCAAACCAACATTGAGGCAGATTATGGCCCACTTT
                   ************************************************************

ClYVV-IA-2016      TCCCAGGCAGCCGAATCATACATAGAGTTTAGGAACTCAACAGAGAAATATATGCCTAGG
ClYVV-IA-2017      TCCCAGGCAGCCGAATCATACATAGAGTTTAGGAACTCAACAGAGAGATACATGCCTAGG
                   ********************************************** *** *********

ClYVV-IA-2016      TATGGGCTGCAAAGAAATCTCACAGACTATGGATTGGCCAGATATGCATTTGACTTCTAC
ClYVV-IA-2017      TATGGACTGCAAAGAAATCTCACAGACTATGGATTGGCCAGATATGCATTTGACTTCTAC
                   ***** ******************************************************

ClYVV-IA-2016      AGGTTGACCTCAAAGACACCAGCAAGAGCTCGAGAAGCACATATGCAAATGAAGGCAGCA
ClYVV-IA-2017      AGGTTGACCTCAAAGACACCAGCAAGAGCTCGAGAAGCACATATGCAAATGAAGGCAGCA
                   ************************************************************

ClYVV-IA-2016      GCAATAAGAGGAAAGTCGAACCACATGTTTGGACTGGATGGCAATGTTGGAACAGACGAG
ClYVV-IA-2017      GCAATAAGAGGAAAGTCAAACCACATGTTTGGACTGGATGGCAATGTTGGAACAGACGAG
                   ***************** ******************************************

ClYVV-IA-2016      GAGAACACAGAAAGGCACACAGCAAATGATGTCAACAGGAACATGCATCACATTGCTGGA
ClYVV-IA-2017      GAGAACACAGAAAGGCACACAGCAAATGATGTCAACAGGAACATGCATCACATTGCTGGA
                   ************************************************************

ClYVV-IA-2016      GCGCGATTCTAGAGTATCCGTCTTTAAATTCTCCGTTAATTTCGAAGTTTTACTATTATA
ClYVV-IA-2017      GCGCGATTCTAGAGTATCCGTCTTTAAATTCTCCGTTAATTTCGAAGTTTTACTATTATA
                   ************************************************************
```

```
ClYVV-IA-2016      GCACTATGTTAAGTGAGGTTTTACCTCCATTTCATTTTAAGTATAATATGTAATCCATTC
ClYVV-IA-2017      GCACTATGTTAAGTGAGGTTTTACCTCCATTTCATTTTAAGTATAATATGTAATCCATTC
                   ************************************************************

ClYVV-IA-2016      TCTCTATTCTGACAGAGTAGCTAAGTGAGGTTATACCTCGTTGTGAATCTGATCTTTATA
ClYVV-IA-2017      TCTCTATTCTGACAGAGTAACTAAGTGAGGTTATACCTCGTTGTGAATCTGATCTTTATA
                   ******************* ****************************************

ClYVV-IA-2016      GAGCGAGAAA--------------------------------------------------
ClYVV-IA-2017      GAGCGAGAAAAAAAATAAAAAATAAATAAAAATCAACCCTTTTTTGTAAATCCCCACCTTT
                   **********

ClYVV-IA-2016      ---------
ClYVV-IA-2017      TGTTCTGCT
```
