# Supplementary material for: Detection and discovery of plant viruses in soybean by metagenomic sequencing
Source: Virol J. 2022 Sep 13;19:149. doi: 10.1186/s12985-022-01872-5 (PMC9472442; doi:10.1186/s12985-022-01872-5)
Supplement: Supplementary file 16 — Additional file 16 Table S6. Systemic responses of host genotypes to inoculation with soybean mosaic virus-N (N), clover yellow vein virus-No.30 (No.30), HC-Pro derived chimeric N or ClYVV-No.30a [file 12985_2022_1872_MOESM16_ESM.doc]

**Table S6.** Systemic responses of host genotypes to inoculation with soybean mosaic virus-N (N)*,* clover yellow vein virus-No.30 (No.30), HC-Pro derived chimeric N or ClYVV-No.30^a^

**Host genotype**

***Glycine max*** ***Vicia faba***

**Inoculum** (Acre Edge 22R269) (Windsor)

N 3^a^/3 ND^b^

No.30 0/26 4/4

No.30/N HC-Pro 0/22 8/11

N/No.30 HC-Pro 10/13 ND

^a^Total number of plants systemically infected/total numbers of plants mechanically inoculated on primary leaves with sap extract from infected tissues or biolistically inoculated with infectious cDNA clones. Plants were evaluated for presence of systemic symptoms at 21-28 days post-inoculation. Asymptomatic plants were assayed by RT-PCR or back-inoculation on susceptible hosts.

^b^Not done.

It should be noted that infection of soybean with SMV-N or N/ClYVV-No.30 and broadbean with No.30 or No.30/N HC-Pro resulted in expression of symptoms typical of those of parental viruses.
